# Comparing heritability estimators under alternative structures of linkage disequilibrium

**DOI:** 10.1093/g3journal/jkac134

**Published:** 2022-06-08

**Authors:** Alan Min, Elizabeth Thompson, Saonli Basu

**Affiliations:** Department of Statistics, University of Washington, Seattle, WA 98195, USA; Department of Statistics, University of Washington, Seattle, WA 98195, USA; Division of Biostatistics, University of Minnesota, Minneapolis, MN 55455, USA

**Keywords:** Haseman–Elston regression, heritability estimation, linkage disequilibrium, fixed-SNP-effects model, random-SNP-effects model

## Abstract

The single nucleotide polymorphism heritability of a trait is the proportion of its variance explained by the additive effects of the genome-wide single nucleotide polymorphisms. The existing approaches to estimate single nucleotide polymorphism heritability can be broadly classified into 2 categories. One set of approaches models the single nucleotide polymorphism effects as fixed effects and the other treats the single nucleotide polymorphism effects as random effects. These methods make certain assumptions about the dependency among individuals (familial relationship) as well as the dependency among markers (linkage disequilibrium) to provide consistent estimates of single nucleotide polymorphism heritability as the number of individuals increases. While various approaches have been proposed to account for such dependencies, it remains unclear which estimates reported in the literature are more robust against various model misspecifications. Here, we investigate the impact of different structures of linkage disequilibrium and familial relatedness on heritability estimation. We show that the performance of different methods for heritability estimation depends heavily on the structure of the underlying pattern of linkage disequilibrium and the degree of relatedness among sampled individuals. Moreover, we establish the equivalence between the 2 method-of-moments estimators, one using a fixed-single nucleotide polymorphism-effects approach, and another using a random-single nucleotide polymorphism-effects approach.

## Introduction

Fundamental to the study of inheritance is the partitioning of the total phenotypic variation into genetic and environmental components (Visscher *et al.* 2008). Using family studies, the phenotypic variance–covariance matrix can be parameterized to include the variance of an additive genetic effect, and an environmental effect (Lynch and Walsh 1998). Specific family designs, such as twin studies can accommodate both shared and nonshared environmental effects. The ratio of the genetic variance component to the total phenotypic variance is the proportion of genetically controlled variation and is termed as the “narrow-sense heritability.” As shown in the recent review of more than 17,000 twin studies (Polderman *et al.* 2015), heritability provides useful information on the power to identify causal genetic markers in a genome-wide association study (GWAS), is used to estimate familial recurrence risk of disease, and informs the genetic architecture of the trait (e.g. through partitioning by genomic region or tissue-specific expression).

GWASs seek to understand the relationship between these traits and millions of single nucleotide polymorphisms (SNPs), a type of genetic variant. Linear models are widely used in the field of statistical genetics to assess both individual and cumulative contribution of genetic variants on a trait. The individual contribution is assessed by treating each variant as a fixed effect (fixed-SNP-effect model) while adjusting for relevant covariates in a linear regression (Dicker 2014; Bulik-Sullivan *et al.* 2015; Schwartzman *et al.* 2019) or by treating each variant as a random effect (random-SNP-effect model) by using a linear mixed effect model (Yang *et al.* 2010, 2011; Speed *et al.* 2012). The fixed-SNP-effect-based approaches model individuals as independent, but incorporate the dependencies among the markers explicitly into the model. On the other hand, the random-SNP-effect models use the genetic relatedness among individuals to improve the efficiency of estimation of genetic variance. Nowadays, with the increasing ability to sequence many genetic variants in large cohort studies [UK Biobank Bycroft *et al.* (2018), Precision Medicine cohort Collins and Varmus (2015), and the Million Veterans Program Gaziano *et al.* (2016) are a few such examples], there is significant interest to estimate the cumulative contribution of the genome-wide causal variants. Often we assess such cumulative contribution by estimating the proportion of variance explained by the additive effects of the causal variants in the genome; that is, the “SNP heritability.”

The random-SNP-effect models assume an infinitesimal model for the SNP effects and use of genome-wide SNP data on distantly related individuals (Haseman and Elston 1972; Yang *et al.* 2010, 2011, 2012; Lee *et al.* 2011, 2012; Speed *et al.* 2012; Bulik-Sullivan *et al.* 2015; Seal *et al.* 2022) to estimate the pairwise genetic relatedness between sampled individuals. These approaches assume that each causal SNP makes a random contribution to the phenotype, and these contributions are correlated between individuals who have similar genotypes. By partitioning the phenotypic covariance matrix among all individuals into a genetic similarity matrix and a random variation matrix, the approach estimates the proportional contribution of the genetics to the total phenotypic variation. The estimation of the heritability parameter heavily depends on the estimation of a high-dimensional genetic relationship matrix (GRM). The matrix is usually estimated from the observed data on *M* markers for all *n* individuals in the cohort. Two methods of estimation are used to estimate heritability under this model. One is a likelihood-based approach, which includes Genome Wide Complex Trait Analysis, or GCTA (Yang *et al.* 2010) and Linkage-Disequilibrium Adjusted Kinships, or LDAK (Speed *et al.* 2012). It uses the restricted maximum likelihood (REML) estimation technique (Corbeil and Searle 1976) to estimate heritability. The other approach uses method-of-moments (MOM) technique to estimate heritability, such as Haseman–Elston (HE) regression (Haseman and Elston 1972). A major advantage of this mixed effect model approach is that it can account for related individuals, but the general recommendation is to exclude individuals with relatedness greater than 0.025 in the estimation of heritability (Yang *et al.* 2011) due to shared environment. These approaches do not explicitly account for the linkage disequilibrium (LD) among the markers, and REML-based estimators have been shown to be sensitive to the patterns of LD (Speed *et al.* 2012).

There have been attempts to rectify such bias due to LD by partitioning the genome into regions with different LD structures and by assigning a different genetic variance parameter to each partition (Evans *et al.* 2018). Such correction has been shown to improve the bias in heritability estimation for REML-based estimators. However, such corrections are often ad hoc and the performance depends on the underlying LD structure. Recently, Pazokitoroudi *et al.* (2020) used a similar partitioning strategy on the HE regression estimator. However, it is not clear if LD will impact the MOM estimator in the same way as it does the REML-based estimators. Moreover, the performance of the MOM estimators in presence of LD has not been studied extensively.

The fixed-SNP-effect approaches assume SNP effects are arbitrary and fixed (Dicker 2014; Schwartzman *et al.* 2019), thus giving more flexibility to each SNP effect. The proposed estimators are consistent and asymptotically normal in high-dimensional linear models with Gaussian predictors and errors, where the number of causal predictors *m* is proportional to the number of observations *n*; in fact, consistency holds even in settings where m/n→ρ, where 0<ρ<∞. This set of approaches cannot easily accommodate relatives in the model, and thus the consistency of the estimator is derived under the assumption that the sampled individuals have independent genotypes. These approaches directly incorporate the LD among SNPs into the model and have been shown to provide consistent estimates of heritability under the correctly specified LD model for *n *>* M,* where *M* is the total number of markers. However, these methods make different approximations to derive the heritability estimator for *n *<* M*, since the LD matrix is not invertible then. The properties and differences between these approximation-based estimators (Dicker 2014; Hou *et al.* 2019) are not well studied for *n *<* M*.

In this article, we take a closer look at these random-SNP-effects and fixed-SNP-effects models for heritability estimation using both likelihood and MOM approaches. This article provides an analytical comparison of 2 popular MOM estimators from each of these categories. We aim to understand the fundamental differences or similarities between the principles of these 2 lines of approaches. We present a set of simulation studies with varying structures of LD and compare the performance of a wide array of estimators. We further provide some theoretical results that justify the observed simulation performance. We demonstrate through theoretical derivations as well as simulation studies that the potential impact of LD on a random-SNP-effect model-based estimator (Haseman and Elston 1972) depends on the extent and structure of correlation of the causal and noncausal variants. We also show that the fixed-SNP-effect model estimator proposed by Dicker (2014) is essentially equivalent to the HE method-of-moments estimator (Haseman and Elston 1972) for *n *<* m*.

Our findings in this article do not demonstrate any particular advantage of the fixed-SNP-effect models over the random-SNP-effect models in presence of LD, at least for the case when heritability is estimated using a genome-wide marker model and when *n *<* M*. One could partition the genome into small segments to account for the differences in genome-wide LD structure and handle the influence of LD better using a fixed-SNP-effect estimator for each partition separately (Hou *et al.* 2019). However, there is a potential overfitting issue for having a separate heritability parameter for every partitioned segment.

The rest of the article is organized as follows: first, different methods to estimate heritability are explained and analytical formulae to compare their performance under different LD structures are presented. We then describe strategies to simulate LD and relatedness structure and to evaluate both fixed-SNP-effects and random-SNP-effects models. Finally, the results are presented and discussed.

## Materials and methods

### Genotypes, phenotypes, and heritability estimation

We consider a set of *n* individuals from a homogeneous population, typed at *M* SNP markers, assumed to be in Hardy–Weinberg equilibrium. Note that notation is also listed in Table 1. Assume an *n* × *M* matrix of genotypes $G=(G_{ij})$, where $G_{ij}=0,1,2$ is the number of copies of the reference allele for individual *i* at locus *j* with population frequency *p_j_*. Thus $G_{ij},i=1,2,\ldots ,n$, has mean $2p_{j}$ and variance $2p_{j}(1-p_{j}),\;j=1,2,\ldots ,M$. The vector of standardized genotypes for individual *i* at marker *j* is given by

(1) $$\Gamma_{ij}=\frac{G_{ij}-2p_{j}}{\sqrt{2p_{j}(1-p_{j})}},$$

so that Γ_*ij*_ has mean 0 and variance 1.

**Table 1.** Glossary of notation used.

Variable	Definition
*n*	Number of individuals in an analysis
*M*	Total number of markers in an analysis
*m*	The number of causal markers in an analysis. A marker is considered causal if it has a nonzero direct effect on phenotype.
i, k	Typically used to index individuals, i,k=1,…,n.
j, ℓ	Typically used to index markers, j,ℓ=1,…,m or *M*.
*p_j_*	The population frequency of locus *j*
$\sigma_{g}^{2}$	The variance in phenotype attributable to genotypic effects
$\sigma_{e}^{2}$	The variance in phenotype attributable to environment.
*r_jl_*	The genotypic correlation between loci *j* and *l* in the population
$\phi_{ik}$	Genotypic correlation between individuals *i* and *k*
G	A matrix of genotypes with *n* rows and *M* columns
$\Gamma_{A}$	A matrix of normalized genotypes with *n* rows and *M* columns (Equation 1)
$\Gamma_{C}$	A matrix formed by the *m* columns of $\Gamma_{A}$ that correspond to the causal markers
Ψ	The GRM calculated using all markers; an *n *×* n* matrix. $M^{-1}\Gamma_{A}\;\Gamma_{A}^{\prime }$.
Σ	The *M *×* M* marker LD matrix calculated from all markers. $n^{-1}\Gamma_{A}^{\prime }\;\Gamma_{A}$
$\Sigma^{*}$	The true *M *×* M* marker LD matrix calculated from all markers. $E(n^{-1}\Gamma_{A}^{\prime }\;\Gamma_{A})$
β	An *m*-vector of effects of causal loci on phenotype, or sometimes an M-vector augmented by 0’s (Equation 4)
y	An *n*-vector of phenotypic values of individuals.

The matrix of standardized genotypes for all markers, $\Gamma_{A}=(\Gamma_{ij})$, carries information on the relatedness of individuals, and the LD among markers. While $E(\Gamma_{ij}\Gamma_{i\ell })=r_{j\ell }$ is the genotypic correlation between loci within an individual, $E(\Gamma_{ij}\Gamma_{kj})=\phi_{ik}$ measures the genotypic correlation between individuals. We define the GRM Ψ as in Yang *et al.* (2010)

(2) $$\Psi =M^{-1}\Gamma_{A}\;\Gamma_{A}^{\prime }$$

and we define the LD matrix as

(3) $$\Sigma =n^{-1}\Gamma_{A}^{\prime }\;\Gamma_{A}.$$

where we use the single quote (′) to denote the transpose of a matrix. In large samples, the empirical allele frequencies ${\left(2n\right)}^{-1}\sum_{i=1}^{n}G_{ij}$ can be used as an estimate of the population frequency *p_j_* of Equation (1) in forming the matrices Ψ and Σ.

Suppose that the first *m* of the *M* markers are *causal*, having a direct impact on phenotype. We denote by $\Gamma_{C}$ the matrix consisting of the *m* columns of $\Gamma_{A}$ corresponding to the causal markers, and adopt the classical trait model of Fisher (1919). The phenotype of individual *i* is given by

(4) $$y_{i}=\sum_{j=1}^{m}\Gamma_{ij}\beta_{j}+\epsilon_{i},$$

where *β_j_*, Γ_*ij*_, and *ϵ_i_* are mutually independent and have mean 0. Then $E(y_{i}|\Gamma_{C})\;\equiv \;0$ so that $\text{var}(y_{i})=E(\text{var}(y_{i}\;|\;\Gamma_{C}))$. In SNP heritability estimation, $\sigma_{g}^{2}$ is the phenotypic variance attributable to SNPs, and $\sigma_{e}^{2}$ is the phenotypic variance attributable to environmental factors. In a random-SNP-effect model, we assume $\beta_{j}∼N(0,\sigma_{g}^{2}/m)$, and $\epsilon_{i}∼N(0,\sigma_{e}^{2})$. Then

$$\text{var}(y_{i}\;|\;\Gamma_{C})=\text{var}(\sum_{j=1}^{m}\Gamma_{ij}\beta_{j}+\epsilon_{i})=\sum_{j=1}^{m}\Gamma_{ij}^{2}\text{var}(\beta_{j})+\text{var}(\epsilon_{i})=\frac{\sigma_{g}^{2}}{m}\sum_{j=1}^{m}\Gamma_{ij}^{2}+\sigma_{e}^{2}.$$

Then either conditionally on $\Gamma_{C}$ as *m* becomes large, or taking expectations over Γ_*ij*_, $\text{var}(y_{i})=\sigma_{g}^{2}+\sigma_{e}^{2}$. On the other hand, a fixed-SNP-effect model assumes that β is a fixed quantity, with $\beta_{j}=0$ for noncausal markers. In that case, we define $\sigma_{g}^{2}=\beta \prime \Sigma^{*}\beta$, and note $E(\Gamma_{ij}\Gamma_{i\ell })=r_{j\ell }$ and because of normalization, $E(\Gamma_{ij}^{2})=1$ so that

$$\text{var}(y_{i})=\text{var}(\sum_{j=1}^{m}\Gamma_{ij}\beta_{j}+\epsilon_{i})=\sum_{j=1}^{m}\sum_{\ell =1}^{m}\beta_{j}\beta_{\ell }E(\Gamma_{ij}\Gamma_{i\ell })+\text{var}(\epsilon_{i})=\sigma_{g}^{2}+\sigma_{e}^{2}.$$

Thus in either case, the phenotypic variance $\text{var}(y_{i})=\sigma_{g}^{2}+\sigma_{e}^{2}$ and SNP heritability are $h^{2}=\sigma_{g}^{2}/(\sigma_{g}^{2}+\sigma_{e}^{2})$. If phenotypes are standardized to have variance 1, then $\sigma_{g}^{2}=h^{2}$ and $\sigma_{e}^{2}=1-h^{2}$. More generally, estimation of heritability is primarily concerned with the estimation of $\sigma_{g}^{2}$, the estimate of *h*^2^ being then obtained by dividing by the empirical variance of the phenotypes $y_{i},\;i=1,\ldots ,n$.

### Overview of estimators

In our overview of the methodologies for heritability estimation, we concentrate on method-of moments estimation and likelihood-based estimation for the random-SNP-effect models. We further compare these estimators with the fixed-SNP-effect method of moments model-based estimators (Dicker 2014; Schwartzman *et al.* 2019). The Supplementary material provides more details on these estimators.

For the likelihood methods, we consider the GCTA (Yang *et al.* 2011) and LDAK (Speed *et al.* 2012) approaches. In brief, GCTA is a random-SNP-effect model derived under assumptions similar to those of *Genotypes, Phenotypes, and Heritability Estimation*. The approach uses REML (Patterson and Thompson 1971) to estimate $\sigma_{g}^{2}$ and $\sigma_{e}^{2}$. It estimates heritability assuming that phenotypes are drawn from a multivariate Normal distribution, where the log-likelihood function is

(5) $$-\frac{n}{2}\text{log}(2\pi )-\frac{1}{2}(\text{log}\;\det(\sigma_{g}^{2}\Psi +\sigma_{e}^{2}I)+y\prime {\left(\sigma_{g}^{2}\Psi +\sigma_{e}^{2}I\right)}^{-1}y).$$

LDAK (Speed *et al.* 2012) uses a similar model, except reweighting the SNP markers to adjust for LD. More details on the GCTA and LDAK approaches can be found in Supplementary Section 1. Note that $\sigma_{g}^{2}$ is only identifiable when Ψ is not the identity matrix.

For the method-of-moments estimators, we first considered an HE estimator (Haseman and Elston 1972), an estimator from the random-SNP-effect approach category. The estimator of $\sigma_{g}^{2}$, derived in Supplementary Section 2.1, has the form

(6) $$\tilde{\sigma_{g}^{2}}=\frac{S_{Y\Psi }}{S_{\Psi \Psi }}=\frac{\sum_{k}\sum_{i<k}y_{i}y_{k}\Psi_{ik}}{\sum_{k}\sum_{i<k}\Psi_{ik}^{2}}.$$

An estimate of heritability is then given by dividing by the empirical variance of phenotypes *Y_i_*. Further properties of this estimator in the case of no LD are given in Supplementary Section 2.1.

We also considered 2 method-of moment estimators from the fixed-SNP-effect approach category, which we denote Dicker-1 and Dicker-2 (Dicker 2014). Dicker-1 is applicable in the case of no LD. It is derived and discussed in the Supplementary Section 2.2 and takes the form

(7) $${\tilde{\sigma }}_{g}^{2}={\left(n(n+1)\right)}^{-1}(||\Gamma_{A}^{\prime }y|{|}^{2}-My\prime y)={\left(n(n+1)\right)}^{-1}(My\prime \Psi y-My\prime y).$$

We consider this estimator primarily for comparison with the HE estimator: see Supplementary Section 2.1 and in the section Impact of LD on the Dicker-1 estimator.

Throughout this article, we refer to the estimator in Equation (7) as Dicker-1, but we also present a variant of Dicker-1, which we denote Dicker-1-Σ. In the presence of LD, if Σ is invertible, Dicker-1-Σ is

(8) $$\begin{array}{c} {\tilde{\sigma }}_{g}^{2}={\left(n(n+1)\right)}^{-1}((\Sigma^{-1/2}\Gamma_{A}^{\prime }y)\prime (\Sigma^{-1/2}\Gamma_{A}^{\prime }y)-My\prime y)\\ ={\left(n(n+1)\right)}^{-1}(y\prime \Gamma_{A}\Sigma^{-1}\Gamma_{A}^{\prime }y-My\prime y). \end{array}$$

However, in many cases, Σ is not invertible because *M *>* n*, and hence we do not consider Dicker-1-Σ in our simulations. In these cases, to address the LD, Dicker (2014) derives an estimator which we denote Dicker-2. This estimator uses moments of the trace of the LD matrix Σ to correct for LD, resulting in an estimator of $\sigma_{g}^{2}$

(9) $${\tilde{\sigma }}_{g}^{2}=\frac{\mu_{1}(\Gamma_{A}^{\prime }y)\prime (\Gamma_{A}^{\prime }y)-M\mu_{1}^{2}y\prime y}{n(n+1)\mu_{2}}$$

where

(10) $$\mu_{1}=\frac{tr(\Sigma )}{M}\;\text{and}\;\mu_{2}=\frac{tr(\Sigma^{2})}{M}-\frac{{\left(tr(\Sigma )\right)}^{2}}{Mn}.$$

Further details of the Dicker-2 estimator are given in Supplementary Section 3.3.

### Impact of LD

#### Impact of LD on the HE estimator

In this section, we consider the impact of marker mispecification and marker LD on the numerator and denominator of the HE estimator, and hence on the estimate of $\sigma_{g}^{2}$. We assume unrelated individuals but correlated markers, so that $E(\Gamma_{ij}\Gamma_{k\ell })=0$ if i≠k, but $E(\Gamma_{ij}\Gamma_{i\ell })=r_{j\ell }$, with $-1\leq r_{j\ell }\leq 1$, and *r_jj_* = 1.

We split the markers into *m* causal markers *C* and (M−m) noncausal markers *F*. Note that all markers are used in the GRM: $\Psi =M^{-1}\Gamma_{A}\;\Gamma_{A}^{\prime }$, but that only causal markers $\Gamma_{C}$ contribute to the phenotype **y**. For convenience, assume that the first *m* markers are causal: C={1,…,m} and F={(m+1),…,M}. Then, following the same derivation as in Supplementary Section 2.1, for i≠k we obtain,

(11) $$E(y_{i}\Psi_{ik}y_{k})=M^{-1}E\left(\sum_{j=1}^{m}\sum_{\ell =1}^{m}\beta_{j}\beta_{\ell }(\sum_{w=1}^{M}\Gamma_{ij}\Gamma_{iw}\Gamma_{kw}\Gamma_{k\ell })\right).$$

If the *β_j_* have mean 0 and are uncorrelated, we have only terms in j=ℓ, and this reduces to

$$\begin{array}{c} E(y_{i}\Psi_{ik}y_{k})=M^{-1}E\left(\sum_{j=1}^{m}\beta_{j}^{2}(\sum_{w=1}^{M}\Gamma_{ij}\Gamma_{iw}\Gamma_{kw}\Gamma_{kj})\right)\\ =M^{-1}E\left(\sum_{j=1}^{m}\beta_{j}^{2}(\sum_{w=1}^{M}r_{jw}^{2})\right)\\ ={\left(mM\right)}^{-1}\sigma_{g}^{2}\sum_{j=1}^{m}\sum_{w=1}^{M}r_{jw}^{2}. \end{array}$$

Here, using that individuals *i* and *k* are independent and that $\beta_{j}^{2}$ has expectation $\sigma_{g}^{2}/m$. Then

(12) $$\begin{array}{c} E(S_{Y\Psi })=E(\sum \sum_{i<k}y_{i}\Psi_{ik}y_{k})=(n(n-1)/2)E(y_{i}\Psi_{ik}y_{k})\\ =\frac{n(n-1)\sigma_{g}^{2}}{2mM}\sum_{j=1}^{m}\sum_{w=1}^{M}r_{jw}^{2}=\frac{n(n-1)\sigma_{g}^{2}}{2mM}(R_{CC}+R_{CF}), \end{array}$$

where for convenience, we denote the sums of squared correlations

$$\begin{array}{c} R_{CC}=\sum_{j=1}^{m}\sum_{\ell =1}^{m}r_{j\ell }^{2}\;\;\;\text{among causal markers}\\ R_{CF}=\sum_{j=1}^{m}\sum_{\ell =m+1}^{M}r_{j\ell }^{2}\;\text{between causal and non-causal markers}\\ \;\text{and}\;R_{FF}=\sum_{j=m+1\;}^{M}\sum_{\ell =m+1}^{M}r_{j\ell }^{2}\;\;\text{among non-causal markers}. \end{array}$$

Considering similarly the denominator of the HE estimator,

$$E(\Psi_{ik}^{2})=M^{-2}\sum_{j=1}^{M}\sum_{\ell =1}^{M}E(\Gamma_{ij}\Gamma_{kj}\Gamma_{i\ell }\Gamma_{k\ell }))=M^{-2}\sum_{j=1}^{M}\sum_{\ell =1}^{M}r_{j\ell }^{2}$$

so that

$$E(S_{\Psi \Psi })=\sum \sum_{i<k}E(\Psi_{ik}^{2})=\frac{n(n-1)}{2M^{2}}(R_{CC}+2\;R_{CF}+R_{FF})$$

leading finally to the ratio of expectations of $S_{Y\Psi }$ and $S_{\Psi \Psi }$

(13) $$\frac{M}{m}\sigma_{g}^{2}\;\frac{R_{CC}+R_{CF}}{R_{CC}+2\;R_{CF}+R_{FF}}.$$

Equation (13) approximates the expectation of the HE estimator and gives insight into its bias. First, if there is no LD, $R_{CC}=m$, *R_CF_* = 0, and $R_{FF}=(M-m)$, giving the results of Supplementary Section 2. Second, if the GRM contains only causal markers *M *=* m*, then LD among these causal markers does not cause bias, as approximated by Equation (13). Third, if additional markers *F* are not in LD with each other, nor with the causal markers *C*, $R_{CF}=R_{FF}=0$, and again no bias results. Note that generally inclusion of additional markers in the GRM is less serious than omission of causal markers. If $\Gamma_{A}$ is missing causal markers *j*, then Equation (11) will not include the contributions of those *β_j_* and $S_{Y\Psi }$ will be decreased, but $S_{\Psi \Psi }$ will not (on average) be affected, leading to underestimation of $\sigma_{g}^{2}$.

**Fig. 1.**
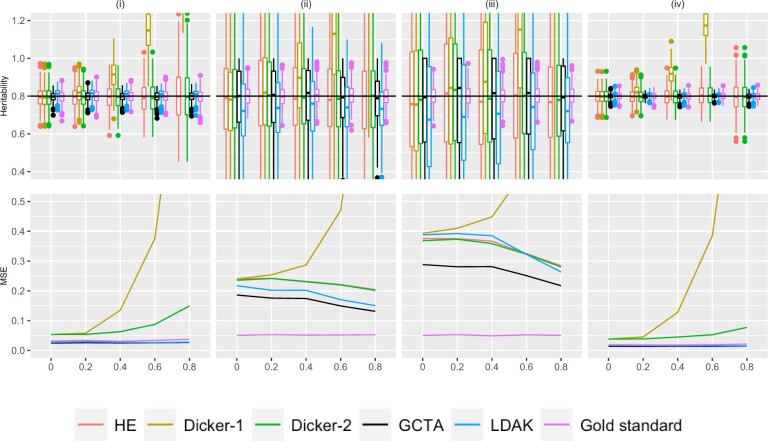
Simulation Study 1A (autocorrelated markers). On the top row, the *X*-axis plots the parameter *ρ*, the autocorrelation correlation coefficient between simulated markers as described in Supplementary Section 4. Estimates of *h*^2^ using different estimators are plotted along the *Y*-axis. The value *n* refers to the number of individuals simulated. The value *M* is the total number of markers simulated, where half of the markers are causal, set in an alternating fashion, as described in *Simulation Strategy*. We consider (i) n=1,000,m=100 (ii) n=200,m=500, (iii) n=200,m=1,500, and (iv) n=2,000,m=500. Five hundred data sets were simulated for each condition. A horizontal line is shown at $h^{2}=.8$, the simulated truth. On the bottom row, the *X*-axis is the parameter *ρ*, and the MSE of each of the estimators is plotted on the *Y*-axis.

**Fig. 2.**
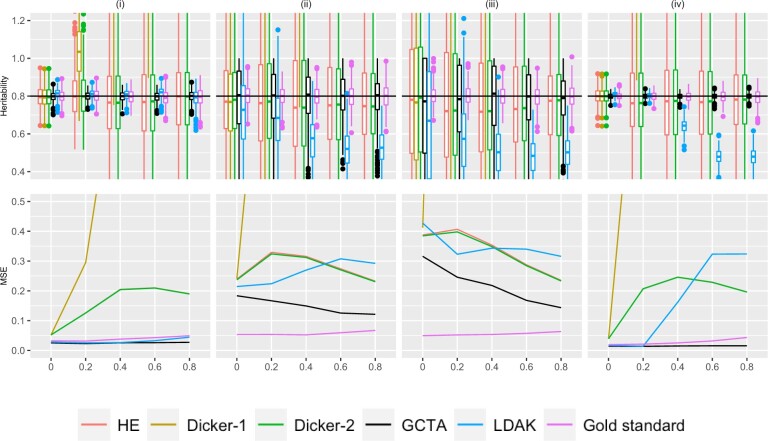
Simulation Study 1B (block markers). On the top row, the *X*-axis plots the parameter *ρ*, the block correlation coefficient between simulated markers as described in Supplementary Section 4. Estimates of *h*^2^ using different estimators are plotted along the *Y*-axis. The value *n* refers to the number of individuals simulated. The value *M* is the total number of markers simulated, where half of the markers are causal, set in an alternating fashion, as described in *Simulation Strategy*. We consider (i) n=1,000,m=100 (ii) n=200,m=500, (iii) n=200,m=1,500, and (iv) n=2,000,m=500. Five hundred data sets were simulated for each condition. A horizontal line is shown at $h^{2}=.8$, the simulated truth. On the bottom row, the *X*-axis is the parameter *ρ*, and the MSE of each of the estimators is plotted on the *Y*-axis.

In some special cases, biases cancel out. Consider first a special case of causal markers in regions of “average LD”; suppose all $r_{j\ell }=s$ for j≠ℓ. Then, $R_{CC}=m+m(m-1)s^{2},\;R_{CF}=m(M-m)s^{2}$ and $R_{FF}=(M-m)+(M-m)(M-m-1)s^{2}$, and some arithmetic show there is no bias. Two other examples occur in the simulations of *Results*. In both the autocorrelation and block simulations, causal and noncausal markers are alternating. Then M=2m and *R_FF_* = *R_CC_*, and Equation (13) again shows there is no bias. This is demonstrated in the simulation results in Figs. 1 and 2. We note that although we only show the case of M=2m, we show in Supplementary Section 3.1 that the approximate theoretical bias is also quite small for other ratios of causal to noncausal markers, and there is no bias for equally sized blocks.

In other cases, there can be bias. For example, if causal markers are in regions of high LD, then (per marker) *R_CC_* dominates over *R_FF_*, and $\sigma_{g}^{2}$ will be overestimated, while if causal markers are in regions of low LD *R_FF_* in the denominator will dominate, and $\sigma_{g}^{2}$ will be underestimated. The case of duplication of markers also considered in the simulations (*Simulation Study 1: Impact of Marker LD*) is different, and Equation (13) again provides an estimate of the bias. In this example, there is no LD in the *m* causal markers, so $R_{CC}=m$. The genotypes at subset of *d* of these markers are replicated *r* additional times, but these replicates are noncausal. So M=m+rd. $R_{CF}=rd$ and $R_{FF}=r^{2}d$. Then Equation (13) reduces to

(14) $$\frac{M}{m}\sigma_{g}^{2}\frac{m+rd}{m+2rd+r^{2}d}=\sigma_{g}^{2}\;\frac{{\left(m+rd\right)}^{2}}{m(m+rd(2+r))}.$$

Note that if no markers are replicated (*d *=* *0) or all markers are replicated (*d *=* m*) then there is no bias. Note also that the result only depends on the proportion of markers replicated. If *d* = *gm*, then Equation (14) reduces to ${\left(1+rg\right)}^{2}\sigma_{a}^{2}/(1+2rg+r^{2}g)$. Although the expectation of the ratio is approximated by the ratio of expectations in Equation (13), simulation shows this approximation gives an accurate estimate of the bias: see Supplementary Section 3.1 (Supplementary Fig. 3).

#### Impact of LD on the Dicker-1 estimator

Through our simulations, we found that the Dicker-1 estimator had a generally greater bias than the HE estimator (*Simulation Study 1: Impact of Marker LD*). This is because the Dicker-1 estimator is derived from a linear expression of quadratic forms which is inflated due to LD. On the other hand, the HE estimator is a ratio of quadratic forms, and LD inflates both the numerator and the denominator, which potentially reduces the overall effect of LD. We recall from Equation (7) that the Dicker-1 estimator takes the form ${\tilde{\sigma }}_{g}^{2}={\left(n(n+1)\right)}^{-1}(My\prime \Psi y-My\prime y)$. Through calculations shown in detail in Supplementary Section 3.2, we show that

$$E({\tilde{\sigma }}_{g}^{2})\approx \sigma_{g}^{2}\;{\left(n+1\right)}^{-1}(K+\frac{\left(n+M-2\right)}{m}(R_{CC}+R_{CF})-M).$$

We note that $R_{CC}+R_{CF}\geq m$, and hence, from this approximation, we have that ${\tilde{\sigma }}_{g}^{2}$ is greater than *σ_g_*, and the magnitude to which it is greater increases as *R_CC_* and *R_CF_* increase.

#### Equivalence of HE and Dicker-2

As will be shown in *Simulation Study 1: Impact of Marker LD*, estimates from the Dicker-2 and HE regression were very similar, although Dicker-2 explicitly models LD. Analytically, under certain normalization schemes, the 2 estimators are effectively equivalent. This suggests that efforts to correct for LD in the Dicker-2 framework do not ensure improved performance of this estimator compared to the HE estimator.

We begin by reformulating HE regression. We recall from Equation (6) that the HE estimator is given by

$$\tilde{\sigma_{g}^{2}}=\frac{S_{Y\Psi }}{S_{\Psi \Psi }}=\frac{\sum_{k}\sum_{i<k}y_{i}y_{k}\Psi_{ik}}{\sum_{k}\sum_{i<k}\Psi_{ik}^{2}}.$$

We can rewrite this in matrix form, giving us

$$S_{Y\Psi }=\frac{y\prime \Psi y-y\prime \mathrm{diag}(\Psi )y}{2}.$$

Under Hardy–Weinberg equilibrium, the GRM should have values that are approximately 1 on the diagonal. We assume *y* is normalized to have variance 1, which results in

$$S_{Y\Psi }\approx \frac{y`\Psi y-n}{2}.$$

Now we consider $S_{\Psi \Psi }$. Noting that $tr(\Psi \prime \Psi )=\sum_{i}\sum_{j}\Psi_{ij}^{2}$,

$$\begin{array}{c} S_{\Psi \Psi }=\frac{tr(\Psi \prime \Psi )-\mathrm{diag}(\Psi )\prime \mathrm{diag}(\Psi )}{2}\\ \approx \frac{tr(\Psi \prime \Psi )-n}{2}. \end{array}$$

Together, the HE estimator is approximately

(15) $$\frac{y`\Psi y-n}{tr(\Psi `\Psi )-n}.$$

Now consider the equations for Dicker-2. First note that in Equations (9) and (10), $\mu_{1}=1$ since the genotypes are normalized to have variance 1. Next, we use the property of traces that tr(ABCD)=tr(DABC) to calculate

$$\begin{array}{c} \mu_{2}=\frac{1}{M}tr(\frac{1}{n^{2}}\Gamma_{A}^{\prime }\Gamma_{A}\Gamma_{A}^{\prime }\Gamma_{A})-\frac{1}{Mn}{\left(tr(\frac{1}{n}\Gamma_{A}^{\prime }\Gamma_{A})\right)}^{2}\\ \approx \frac{1}{M}tr(\frac{1}{n^{2}}\Gamma_{A}^{\prime }\Gamma_{A}\Gamma_{A}^{\prime }\Gamma_{A})-\frac{M}{n}\\ =\frac{M}{n^{2}}tr(\frac{1}{m^{2}}\Gamma_{A}\Gamma_{A}^{\prime }\Gamma_{A}\Gamma_{A}^{\prime })-\frac{M}{n}\\ =\frac{M}{n^{2}}tr(\Psi \prime \Psi )-\frac{M}{n}. \end{array}$$

Since n≈n+1, for large *n*, we have that the Dicker 2 estimator (Equation 9) is approximately

$$\frac{y\prime \Gamma_{A}\Gamma_{A}^{\prime }y-My\prime y}{\left(\frac{M}{n^{2}}tr(\Psi \prime \Psi )-\frac{M}{n})(n)(n+1\right)}\approx \frac{y\prime \Psi y-n}{tr(\Psi \prime \Psi )-n},$$

which is the same as Equation (15).

### Impact of relatedness of individuals on moments estimators

Under the assumption of independence of individuals, the SD of the HE estimator of $\sigma_{g}^{2}$ or of *h*^2^ increases with the number of markers *M* (Supplementary Section 2). This arises because in the limit, the matrix Ψ converges in probability to the identity matrix, i.e. all off-diagonal terms converge in probability to 0. This leads to poor behavior of the HE estimator because the numerator and denominator of the HE estimator converge in probability to 0. However, this is an artifact of the assumption of complete independence (unrelatedness) of individuals. In any real sample, regardless of the extent of correction for population structure, there will always be variation in the degree of relatedness of individuals, even if any single pairwise relatedness measure is small. Note that the original formulation of HE estimators (Haseman and Elston 1972) made use of the genetic similarity between known relatives. In this section, we therefore consider the case where individuals may be related, so standardized genotypes Γ_*ij*_ and Γ_*kj*_ are no longer independent. For simplicity, we ignore LD: that is Γ_*ij*_ and $\Gamma_{k\ell }$ are independent, for j≠l, whether or not *i *=* k*.

Under relatedness and inbreeding, it remains the case that $E(\Gamma_{ij})=0$, but $\text{var}(\Gamma_{ij})=(1+F_{i})$ (Crow *et al.* 1970) and $E(\Gamma_{ij}\Gamma_{kj})=\phi_{ik}$, where *F_i_* is the inbreeding coefficient of individual *i*, and $\phi_{ik}$ is the relatedness of *i* and *k*, or twice the coefficient of kinship between *i* and *k*. To consider the HE estimator (6), for i≠k,

(16) $$\begin{array}{c} E(\Psi_{ik}^{2})=M^{-2}\sum_{j=1}^{M}\sum_{\ell =1}^{M}E(\Gamma_{ij}\Gamma_{kj}\Gamma_{i\ell }\Gamma_{k\ell })\\ =M^{-2}\sum \sum_{j\neq \ell }E(\Gamma_{ij}\Gamma_{kj}\Gamma_{i\ell }\Gamma_{k\ell })+M^{-2}\sum_{j=1}^{M}E(\Gamma_{ij}^{2}\Gamma_{kj}^{2})\\ =M^{-2}(M(M-1)){\left(E(\Gamma_{ij}\Gamma_{kj})\right)}^{2}+M^{-1}E(\Gamma_{ij}^{2}\Gamma_{kj}^{2}))\\ ={\left(E(\Gamma_{ij}\Gamma_{kj})\right)}^{2}+M^{-1}(E(\Gamma_{ij}^{2}\Gamma_{kj}^{2})-{\left(E(\Gamma_{ij}\Gamma_{kj})\right)}^{2}). \end{array}$$

Hence as $M\to \infty \;S_{\Psi \Psi }$ tends to

$$E(\sum_{k}\sum_{i<k}\Psi_{ik}^{2})\;\to \;\sum_{k}\sum_{i<k}\phi_{ik}^{2}.$$

We can also calculate

$$\begin{array}{c} E(y_{i}y_{k}\Psi_{ik})=\frac{1}{M}E\left[\left(\sum_{\ell =1}^{m}\Gamma_{i\ell }\beta_{\ell }\right)\left(\sum_{w=1}^{m}\Gamma_{kw}\beta_{w}\right)\left(\sum_{j=1}^{M}\Gamma_{ij}\Gamma_{kj}\right)\right]\\ =\frac{1}{M}E\left[\left(\sum_{\ell =1}^{m}\Gamma_{i\ell }\Gamma_{k\ell }\beta_{\ell }^{2}\right)\left(\sum_{j=1}^{M}\Gamma_{ij}\Gamma_{kj}\right)\right]\\ =\frac{\sigma_{g}^{2}}{mM}E\left[\left(\sum_{\ell =1}^{m}\sum_{j=1}^{M}\Gamma_{i\ell }\Gamma_{k\ell }\Gamma_{ij}\Gamma_{kj}\right)\right]\\ =\frac{\sigma_{g}^{2}}{mM}E\left[\sum_{j=1}^{\ell -1}\sum_{\ell =2}^{m}\Gamma_{i\ell }\Gamma_{k\ell }\Gamma_{ij}\Gamma_{kj}+\sum_{j=\ell +1}^{M}\sum_{\ell =1}^{m}\Gamma_{i\ell }\Gamma_{k\ell }\Gamma_{ij}\Gamma_{kj}+\sum_{\ell =1}^{m}\Gamma_{il}^{2}\Gamma_{kl}^{2}\right]\\ =\frac{\sigma_{g}^{2}}{mM}(mM-m){\left(E(\Gamma_{i\ell }\Gamma_{k\ell })\right)}^{2}+\frac{\sigma_{g}^{2}}{M}E(\Gamma_{il}^{2}\Gamma_{kl}^{2})\\ =\sigma_{g}^{2}{\left(E(\Gamma_{i\ell }\Gamma_{k\ell })\right)}^{2}+\frac{\sigma_{g}^{2}}{M}(E(\Gamma_{il}^{2}\Gamma_{kl}^{2})-{\left(E(\Gamma_{i\ell }\Gamma_{k\ell })\right)}^{2}) \end{array}$$

and $S_{Y\Psi }$ tends to

$$E(\sum_{k}\sum_{i<k}y_{i}y_{k}\Psi_{ik})\;\to \;\sigma_{g}^{2}\sum_{k}\sum_{i<k}\phi_{ik}^{2}.$$

Thus, contrary to the results of Supplementary Section 2.1 for unrelated individuals, the SD of the HE estimator no longer increases as M→∞, but rather will depend on the magnitude of $\left(\sum_{k}\sum_{i<k}\phi_{ik}^{2}\right)$. Although this sum may be small, if even any of the $\phi_{ik}$ are nonzero it is strictly positive, and eventually, relatedness will bound the SD of the estimator of $\sigma_{g}^{2}$.

Relatedness poses greater problems for the Dicker-1 estimator (Equation 7), which involves the diagonal terms of the GRM matrix Ψ. Considering the expected quadratic form

$$E(M\;y\prime \Psi y)=\sum_{i=1}^{n}\sum_{k=1}^{n}E\left(\left(\sum_{j=1}^{m}\Gamma_{ij}\beta_{j}+\epsilon_{i})(\sum_{w=1}^{M}\Gamma_{iw}\Gamma_{kw})(\sum_{\ell =1}^{m}\Gamma_{k\ell }\beta_{\ell }+\epsilon_{k}\right)\right).$$

Now, by expanding and simplifying, even the coefficient of $\sigma_{e}^{2}$ is no longer *mn* but $\sum_{i=1}^{n}\sum_{w=1}^{m}E(\Gamma_{iw}^{2})=M(n+\sum_{i=1}^{k}F_{i})$ while that of $\sigma_{g}^{2}$ is, as in Supplementary Section 2.2

$$\begin{array}{c} (M-1)\;E(\sum_{i}\Gamma_{ij}^{2}\Gamma_{iw}^{2})+E(\sum_{i}\sum_{k}\Gamma_{ij}^{2}\Gamma_{kj}^{2})=\\ \;\;\;\;\;\;(M-1)\sum_{i=1}^{n}(E(\Gamma_{ij}{)}^{2}{)}^{2}+\sum_{i=1}^{n}E(\Gamma_{ij}^{4})+\sum_{i}\sum_{k\neq i}E(\Gamma_{ij}^{2}\Gamma_{kj}^{2}). \end{array}$$

This expectation now involves not only ${\left(1+F_{i}\right)}^{2}$, and $\phi_{ik}^{2}$ but also higher-order moments.

Although the derivation of distributional properties of the Dicker method-of-moments estimators depends critically on the assumption of 2*n* independent genomes, there is nothing in the derivation of *Equivalence of HE and Dicker-2* that assumes Ψ is diagonal. Indeed, the trace equation

$$n^{2}\;tr(\Sigma^{2})=tr(\Gamma_{A}^{\prime }\;\Gamma_{A}\;\Gamma_{A}^{\prime }\;\Gamma_{A})=tr(\Gamma_{A}\;\Gamma_{A}^{\prime }\;\Gamma_{A}\;\Gamma_{A}^{\prime })=M^{2}\;tr(\Psi^{2})$$

used in showing the approximate equivalence of the HE and Dicker-2 estimators, suggests that the Dicker-2 accommodation of LD in the absence of relatedness is alternatively accommodating relatedness in the absence of LD. Thus, as will be seen in the results of *Simulation Study 3: Impact of Relatedness in Individuals*, the close equivalence of the Dicker-2 and HE estimators should hold under relatedness, and, as seen from Equation (16) above, the standard deviation will no longer increase indefinitely as M→∞.

### Simulation strategy

We performed simulation studies to assess the impact of LD structure and relatedness of individuals on heritability estimation. Each simulated data set consisted of genotypes **G** at *M* markers (*m* causal markers) for *n* unrelated individuals. The marker allele frequencies were those of a randomly chosen subset of markers from the 1000 Genomes Project from Chromosome 1 in the African (AFR) population (Clarke *et al.*, 2017). This set of frequencies was filtered to have allele frequency less than 0.95 and greater than 0.05 and was fixed over data set simulations.

Genotypes are standardized using their empirical allele frequencies. Phenotypes were simulated for *n* individuals, given their genotypes at the *m* causal markers, in accordance with the linear model of Equation (4):

(17) $$y_{i}=\sum_{j=1}^{m}\Gamma_{ij}\beta_{j}+\epsilon_{i}\;\;\;\;\text{for}\;\;i=1,\ldots ,n.$$

For the chosen value of *h*^2^, $\left(0<h^{2}<1\right)$, the *m*-vector of genetic effects β was simulated with independent components $\beta_{j}∼N(0,h^{2}/m)$ for j=1,…,m. The independent residual effects $\epsilon_{i}∼N(0,1-h^{2})$ for i=1,…,n. Thus, for the purposes of the simulation $\sigma_{g}^{2}=h^{2}$, $\sigma_{e}^{2}=1-h^{2}$, and $\text{var}(y_{i})=1$, with *h*^2^ set to 0.8 for all simulations (see *Genotypes, Phenotypes, and Heritability Estimation*).

We implemented the Dicker and HE estimators in R Version 4.0.2 as described in Supplementary Section 2. We used GCTA (Yang *et al.* 2011) and LDAK (Speed *et al.* 2012) as representative likelihood estimators, both of which are described in more detail in Supplementary Section 1. For every simulated data set, we applied each of these estimators. We also report a gold standard estimator to assess the performance of these different methods. The gold standard estimate is calculated assuming we know the true values of β: the empirical variance of $\Gamma_{C}\beta$ is divided by the empirical variance of the phenotypes. This gold standard estimator can be expressed as

(18) $$\frac{\left(\Gamma_{C}\beta -\bar{\Gamma_{C}\beta })\prime (\Gamma_{C}\beta -\bar{\Gamma_{C}\beta }\right)}{\left(y-\bar{y})\prime (y-\bar{y}\right)}.$$

In *simulation study 1*, we assessed the impact of different LD structures on heritability estimation. We generated genotypes assuming three kinds of LD structure: autocorrelated, block, and repeat. More details of the LD structures are given in Supplementary Section 4. Each data set was simulated with a new β and G. For each LD structure, we studied the impact of both sample size *n* and the number of causal markers *m* on heritability estimation, and simulated data sets at 5 levels of LD. For each LD structure and level, we generated 500 simulated data sets.

For the autocorrelation and block structures, we considered the following combinations of *n* and *m*: (1) n=1,000,m=100, (2) n=200,m=500, (3) n=200,m=1,500, and (4) n=2,000,m=500. Comparing (1) and (2) provides insight on differences in estimates of *h*^2^ depending on if *n *>* m* or *m *>* n*, whereas (2) and (3) compares estimates with different number of causal markers, and (2) and (4) compares estimates with different numbers of individuals. We first generated genotypes at M=2m markers. We used marker correlations ρ=0,0.2,0.4,0.6, and 0.8., as detailed in Supplementary Section 4. (Note that *ρ *= 0 is the no-LD case.) The markers were then assigned to be alternating causal and noncausal (m=M/2).

For the repeat structure, we considered the cases: (1) n=1,000,m=200, (2) n=200,m=1,000, (3) n=200,m=3,000, and (4) n=2,000,m=1,000. In this case, we first simulated genotypes for the *m* independent causal markers. The genotypes at the first 10% of markers were then repeated *r* times, where *r* = 0, 2, 4, 6, or 8. (Note that *r *=* *0 is the no-LD case.) The repeat copies of the markers are noncausal, so the number of noncausal markers is 0.1rm and M=m+0.1rm. In Supplementary Fig. 4, c and f, the first *m* markers are causal, and the last (M−m) are the noncausal repeat copies.

**Fig. 3.**
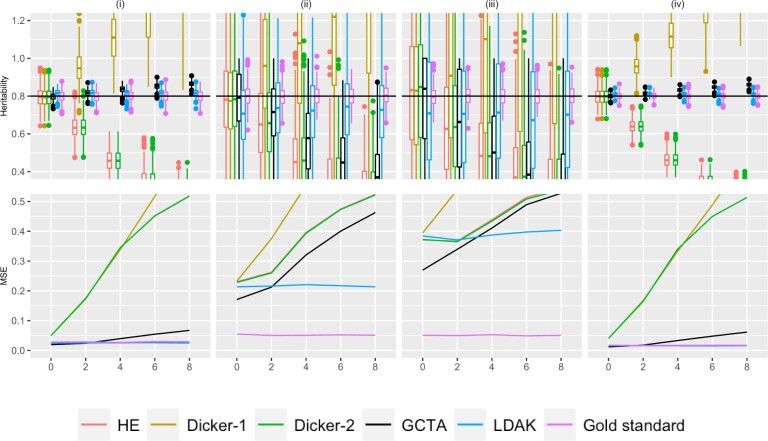
Simulation Study 1C (repeated markers). On the top row, the *X*-axis plots the parameter *r*, the number of times that 10% of the markers are being repeated as described in Supplementary Section 4. Estimates of *h*^2^ using different estimators are plotted along the *Y*-axis. The value *n* refers to the number of individuals simulated. The value *m* is the total number of causal markers simulated, as described in *Simulation Strategy*. We consider (i) n=1,000,m=200 (ii) n=200,m=1,000, (iii) n=200,m=3,000 (iv) n=2,000,m=1,000. Five hundred data sets were simulated for each condition. A horizontal line is shown at $h^{2}=.8$, the simulated truth. On the bottom row, the *X*-axis is the parameter *r*, and the MSE of each of the estimators is plotted on the *Y*-axis.

**Fig. 4.**
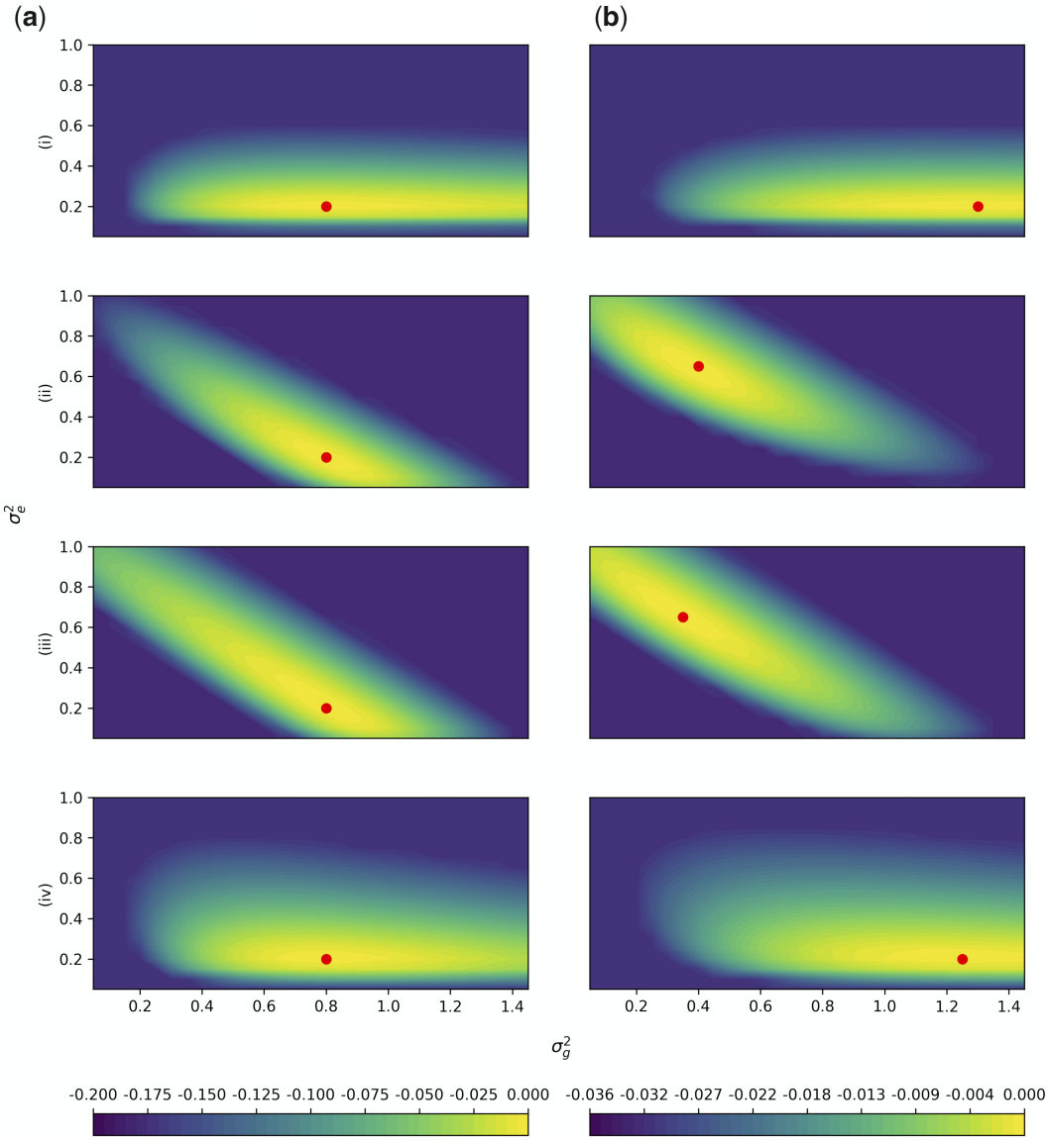
Simulation Study 2. The difference of the log-likelihood from the maximum log-likelihood is plotted for parameters $\sigma_{e}^{2}$ on the *Y*-axis and $\sigma_{g}^{2}$ on the *X*-axis. The colors depict the value of the difference from the maximum log-likelihood. Likelihoods are truncated at the 60% quantile of b(i) for rows (i) and (iv), and at the 60% quantile of a(ii) for rows (ii) and (iii) for visibility. Row labels correspond with Fig. 3, with (i) n=1,000,m=200 (ii) n=200,m=1,000, (iii) n=200,m=3,000, and (iv) n=2,000,m=1,000. Column A has markers with no LD, and in column B, 10% of the markers are repeated 8 times, corresponding the rightmost points in Fig. 3. The average of 100 independent simulations using a grid with spacing 0.05 is plotted in each panel. Note that there is one color scale shared between (i) and (iv) on the left, and a different color scale shared between (ii) and (iii) on the right due to different ranges. The red point indicates the location of the maximum likelihood.

In *simulation study 2*, we investigated the behavior of likelihood models by plotting log-likelihood values (Equation 5) as a function of $\sigma_{g}^{2}$ and $\sigma_{e}^{2}$. The GRM Ψ in Equation (5) was calculated using Equation (2). Of interest was the relationship between the shape of the log-likelihood function and the number of individuals and causal markers, and the shape of the likelihood as the number of repeats increased. From the results of simulation study 1, we hypothesized that the shape would be different when *m *>* n*, where GCTA underestimated heritability, compared to when *m *<* n*, where GCTA overestimated [comparing (i) and (iv) of Fig. 3]. The combinations of numbers of markers and individuals were the same as with the repeats in Simulation Study 1, and the allele frequencies were taken from the AFR sample of the 1000 Genomes Project, as before. We include plots with no repeated markers (Fig. 4a) and with 10% of the markers repeated 8 times (Fig. 4b).

The log-likelihoods minus the maximum log-likelihood were plotted. Likelihoods were truncated at the 60% quantile of b(i) for rows (i) and (iv), and at the 60% quantile of a(ii) for rows (ii) and (iii). These cutoffs were chosen because they were the plots that had the lowest 60% quantile. Plots were generated for (i) n=1,000,m=200 (ii) n=200,m=1,000, (iii) n=200,m=3,000 (iv) n=2,000,m=1,000, in following with simulation study 1. We averaged log-likelihoods of 100 simulated data sets with grid spacing 0.05. Due to differences in ranges, there is a shared color bar between (i) and (iv), and a different shared color bar between (ii) and (iii). A circle within each plot is used to mark the location of the maximum log-likelihood.

In *simulation study 3*, we assessed the impact of related individuals on heritability estimation. We simulated first, second, and third cousins using the rres package in R (Wang *et al.* 2017) as well as unrelated individuals to illustrate our findings in *Impact of Relatedness of Individuals on Moments Estimators*. The segment length option in rres was set to 3,000 cM. Using the same set of allele frequencies as previously, we simulated marker genotypes for 400 individuals, in 10 40-ships. A *k*-ship is defined to be a set of *k* cousins related to a certain degree. Each cousinship is unrelated with all other cousinships. The number of markers ranged from 400 to 4,000 in steps of 400. Phenotypes were generated using Equation (17). For every combination of cousinships and number of markers, we simulated 500 sets of 10 40-ships. A visualization of the GRM of the dataset is shown in Supplementary Fig. 5 using 1,000 markers.

## Results

### Simulation study 1: bias and variance when *ρ* = 0 or *r *=* *0 (no LD)

The special case of no LD in Simulation Study 1 is shown in Figs. 1–3 in panels of the upper row at the left-hand point of each point. These figures verify that the estimators were generally unbiased in estimating the heritability. One exception is in LDAK, where when *n *=* *200, LDAK seemed to underestimate heritability.

Although we generally observed no bias in the estimators under independent markers, we saw that the estimators had a wide range of variances. In the cases n=1,000,M=200 and n=2,000,M=1,000 [columns (i) and (iv) in Figs. 1–3], the variance of the GCTA, and LDAK estimators were lower than the variance of the moments estimators, but this difference is less pronounced in the cases where *n *=* *200 [columns (ii) and (iii)], which may suggest that the number of individuals affects the likelihood-based estimators more than the moments-based estimators. The lower variance resulted in lower MSE for GCTA for all conditions with *ρ* = 0, but the bias in LDAK caused it to have comparable MSE to the moments estimators when *n *=* *200 (Figs. 1–3).

We can also compare cases when the number of individuals is kept constant while the number of markers is increased by comparing n=200,m=500 in column (ii) vs n=200,m=1,500 in column (iii). In Supplementary Section 2.1, we found that with unrelated individuals and independent markers, the standard deviation of heritability should be asymptotically proportional to $\sqrt{M}/n$ in the case of the HE estimator. Accordingly, since M=2m in the simulations, when the number of causal markers increases, the standard deviation of the heritability estimates increased as well. This is shown in both Figs. 1–3, where MSEs were higher in column (iii) compared to column (ii). This trend appeared to hold true for both the likelihood-based estimates and the moments-based estimates.

We can compare cases when the number of individuals increased while holding the number of markers constant by comparing n=200,m=500 in column (i) vs n=2,000,m=500 in column (iv). The variance and MSE of the heritability estimates decreased for all estimators, which agreed with the theoretical result for the HE estimator.

Finally, some of the biases in the behavior of LDAK may be that the LDAK model does not match our generative model. LDAK reweights their genotypes using $X_{ij}=(G_{ij}-2f_{j})\times {\left[2f_{j}(1-f_{j})\right]}^{\alpha }$, and *α* is recommended to be 1.25 (Speed *et al.* 2012, 2017). More details can be found in Supplementary Section 1. Our model does not explicitly simulate phenotypes in this manner, however. To investigate this, we also chose *α* in LDAK to be –1, which matches our simulated phenotypes due to our normalization scheme (Equation 1). Results (not included) were largely similar, although the estimated heritability was slightly closer to the simulated truth in the repeat case.

### Simulation study 1: impact of marker LD

#### Autocorrelation structure

Data were simulated using the autocorrelation structure as described in *Simulation Strategy*, and a representative set of moments and likelihood estimators are evaluated on these simulated data. The estimated variance and bias of different estimators are shown in Fig. 1.

The HE estimator and the Dicker-1 estimator do not explicitly account for LD structure, and because the Dicker-1 estimate was developed for the no-LD case, it shows bias when LD is present. In the top row of Fig. 1, the Dicker-1 estimator shown in gold showed an increase in bias as *ρ* increased for all of (i)–(iv). Consequently, the MSE of the Dicker-1 estimator increases rapidly compared to all of the other estimators as we increase *ρ* (bottom row of Fig. 1). In contrast, there was no increase in the MSE of the HE estimator when markers were autocorrelated, agreeing with *Impact of LD on the HE Estimator*. In the top row of Fig. 1, the estimates of *h*^2^ from the HE estimator did not appear to visually differ significantly from the true value of 0.8. This estimate behaved very similarly to the Dicker-2 estimator, despite the Dicker-2 estimator explicitly attempting to correct for LD. This is analytically shown in *Equivalence of HE and Dicker-2*.

The likelihood estimators in Fig. 1 showed generally lower MSE and no obvious bias. The GCTA estimator is shown in black and the LDAK estimator is shown in light blue. Both of these estimators seemed to have lower MSE across all values of *ρ* than the moments estimators, as seen in the bottom row.

When n=200,m=3,000, as *ρ* increased, there was a decrease in the MSE in all the estimators except the Dicker-1 estimator. In Fig. 1, it can be seen that as *ρ* increases, the first and third quartiles of the estimates of *h*^2^ decrease. It has previously been shown that fewer causal markers lead to decreased variance (Dicker 2014), and hence this effect may be driven by a decrease in the effective number of markers as LD increases.

#### Block structure

Figure 2 shows the estimated variance and bias in different estimators when the genotypes were simulated from the block structure with parameter *ρ*, as described in *Simulation Strategy*. Similarly to the autocorrelation structure, the Dicker-1 estimator had significant bias and high MSE, although this is expected because Dicker-1 as implemented here relates to the no LD case. The HE and Dicker-2 estimators were not as affected by the LD. In contrast to the autocorrelation, however, LDAK underestimated *h*^2^ in Fig. 2, columns (ii), (iii), and (iv). In the bottom row of Fig. 2, this resulted in an MSE that was comparable to that of HE and Dicker-1. GCTA estimates appeared to still largely be unbiased and produced MSEs that were lower than the other estimators. Again, it was observed that there are cases when the MSE decreases as *ρ* increases, similarly to the autocorrelation case.

#### Repeat structure

Figure 3 shows the variance and bias patterns when the genotypes were simulated from the repeat structure with parameter *r*, as described in *Simulation Strategy*. As *r* increases, the number of times that 10% of the markers were simulated increased. There were *m* causal markers simulated and *n* individuals. For example, when *m *=* *1,000 and *r *=* *3, there were 1,000 causal markers that were simulated, and the first 100 markers were repeated 3 times, leading to a total of 1,300 markers that were entered into the analysis. An increased value of *r* indicates more markers that are in perfect LD with the original causal markers. We also examined behavior when repeated markers had a small probability of not being exact duplicates, and results were similar but less pronounced (results not shown).

As in Figs. 1 and 2, the estimates for Dicker-1 increase rapidly as *r* increases, agreeing with analytical calculations from *Impact of LD on the Dicker-1 Estimator*. In contrast to Figs. 1 and 2, in Fig. 3, the estimates for HE and Dicker-2 decrease as *r* increases, corresponding to Equation (14) and to results in Supplementary Fig. 3, where those equations were verified through simulation. The MSE of these 2 estimators also increases as *r* increases (Fig. 3) and further produce very similar estimates, agreeing with analytical calculations from *Equivalence of HE and Dicker-2*.

In Fig. 3, the GCTA estimator produces estimates that are greater than $h^{2}=0.8$ when n=1,000,m=200 and when n=2,000,m=1,000, but produces estimates that are lower than 0.8 when n=200,m=1,000, and when n=200,m=3,000. In other words, if *n *>* m*, then the GCTA estimator is underestimating, and when *n *<* m*, the GCTA estimator is overestimating.

The LDAK estimator shows the same pattern of bias as GCTA in that as *r* increases, *h*^2^ is underestimated when *n *>* m* and overestimated when *n *<* m*. This bias is less pronounced than with GCTA, however. In the bottom panel of Fig. 3, it can be seen that as *r* increases, the MSE of LDAK appears relatively constant, whereas the MSE of GCTA is increasing when n=200,M=1,000 or n=200,M=3,000, as seen in columns (ii) and (iii).

### Simulation study 2: likelihood surfaces

In Fig. 3, GCTA displayed an upward bias when *n *>* m*, and a downward bias when *n *<* m*. We hence hypothesized that the likelihood would be different if *n *>* m* vs if *m *>* n*. The likelihood surface captures the joint likelihood of $\sigma_{e}^{2}$ and $\sigma_{g}^{2}$. From the model in Equation (5), $\mathrm{Var}(y_{i})=\sigma_{e}^{2}+\sigma_{g}^{2}$. Hence we expect that the maximum likelihood lies on a diagonal, as $\sigma_{g}^{2}\approx \mathrm{Var}(y_{i})-\sigma_{e}^{2}$. This appears to be true when the number of individuals is much larger than the number of markers, but when the number of individuals much less than the number of markers, the axis of the conditional maxima becomes more horizontal (Fig. 4). An intuition for this result is that as the number of individuals improves, we have better knowledge of the total phenotypic variance.

The likelihood surfaces also demonstrate a faster rate of change in the likelihood surface when the number of individuals is increased, comparing Fig. 4, a and g where the range of the colors is greater than in Fig. 4, c and e. This observation corresponds with simulation study 1, where as the number of individuals increased, the variance of the estimates of heritability decreased. Finally, on the right hand side of Fig. 4, the surfaces are still either diagonal or horizontal, but the maxima (circles within each plot) are shifted. This agrees with simulation study 1 results, where there was bias in the GCTA method when the number of repeats increased.

### Simulation study 3: impact of relatedness in individuals

In simulation study 3, we studied the effect of familial structure on estimates of heritability using cousinships and found that an increase in the number of causal markers generally increased MSE unless relatedness was high.

**Fig. 5.**
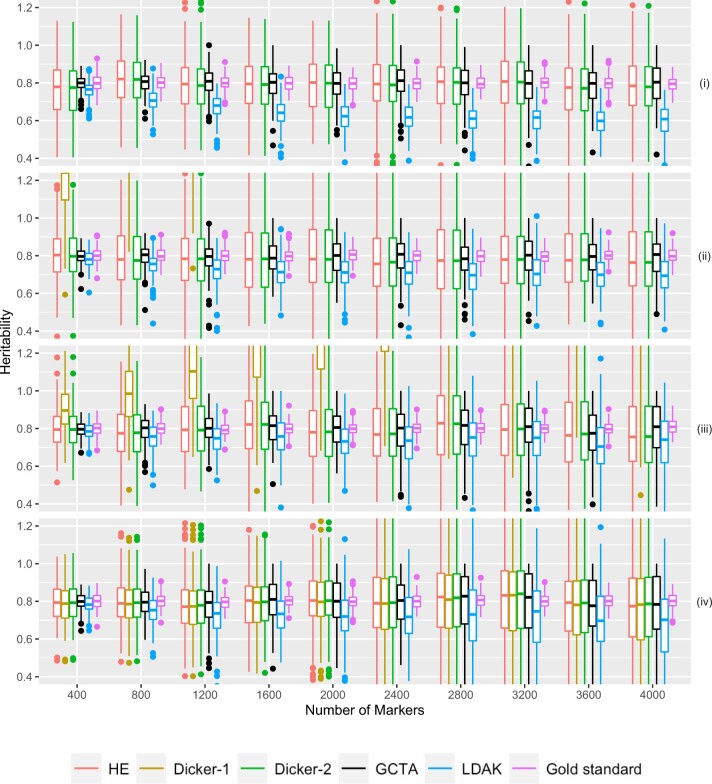
Simulation Study 3. Estimated *h*^2^ from 500 sets of 10 groups of 40 related cousins plotted on the *Y*-axis. The number of causal markers plotted on the *X*-axis. Data were simulated as described in *Simulation Study 3: Impact of Relatedness in Individuals*. Different estimators are plotted in different colors. True heritability was set to be 0.8. Note that because of the chosen range of *y* values, Dicker-1 is sometimes not visible in the figure. Panels (i), (ii), (iii), and (iv) are first-, second-, third-cousins, and unrelated individuals, respectively.

The Dicker-2, HE, and GCTA estimators appeared unbiased for each of the relatedness structures (Fig. 5). For GCTA and HE, we reasoned that because their model is conditional on the GRM, it took into account relatedness. Furthermore, because we have shown that HE and Dicker-2 are equivalent (*Equivalence of HE and Dicker-2*), we can also explain the unbiasedness of Dicker-2. LDAK was also largely unbiased in the case of unrelated individuals, but in the case of first cousins, as the number of markers increased, we observed that LDAK started showing downward bias.

**Fig. 6.**
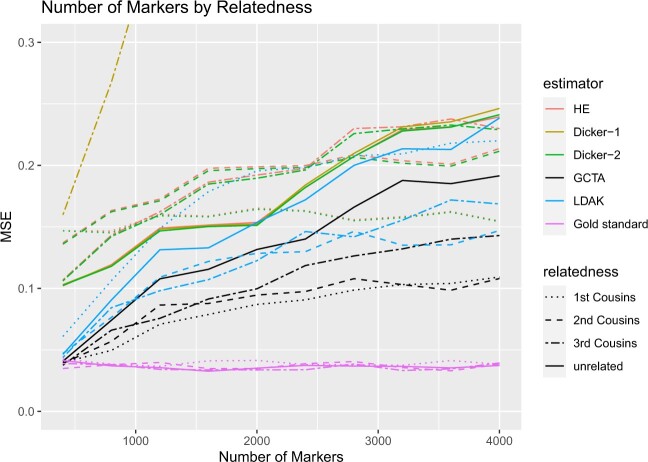
Simulation Study 3. MSE of estimates of *h*^2^ from Fig. 5. The *X*-axis indicates the number of markers in the simulation and the *Y*-axis indicates the mean square error.

For the different relatedness structures (unrelated, full sibs, first cousins) we considered, we observed similar pattern in the change of MSE as we increased the number of markers. MSE was generally the lowest when the number of markers was closer to the sample size. However, as we increased the number of markers, MSE for each estimator increased (Fig. 6). For HE and Dicker-2, the unrelated individuals had the lowest MSE when the number of markers was 400, but increased as the number of markers increased. On the other hand, first cousins had MSE that remained steady (Fig. 6). When the number of markers was 4,000, the MSE of the unrelated individuals was larger than the MSE of the first cousins, agreeing with analytical calculations from *Impact of Relatedness of Individuals on Moments Estimators*.

For each of our estimators, unrelated individuals had the highest MSE and first cousins had the lowest MSE. Furthermore, comparing the case of related to unrelated individuals, the MSE increased more slowly with the increase in the number of causal markers in related individuals.

## Discussion

The methods for SNP heritability estimation can be broadly classified into 2 groups; fixed-SNP-effects models and the random-SNP-effects models. The fixed-SNP-effect models (Dicker 2014; Schwartzman *et al.* 2019) can more easily accommodate the LD structure among the genetic variants and can accommodate variants as both causal or noncausal. However, these approaches rely on independence among the individuals in the sample. On the other hand, the random-SNP-effect models (Yang *et al.* 2011) can accommodate and borrow power from related individuals, though it is generally recommended to exclude relationships with higher relatedness than 0.025 (this corresponds approximately to relatives second cousins or closer) to avoid confounding due to shared environments. These random-SNP-effects models assume all variants are causal and the majority of the methods do not accommodate LD among the markers in a statistically rigorous way. The asymptotic properties of these heritability estimators depend on model assumptions. In this article, we have studied the impact of model misspecification on heritability estimation through extensive simulation studies. We have simulated data under various LD structures and have allowed a certain portion of the variants to be noncausal. We found little difference in the performance of a fixed-SNP-effect model method-of-moments estimator and an MOM estimator from a random-SNP-effect model under different model misspecification.

We have derived the analytic expression for the approximate bias of the HE estimator in presence of LD among markers. *Impact of Linkage Disequilibrium* considers various scenarios for the LD among causal and noncausal markers and analytically shows the impact of this correlation on the HE estimator. Our simulation studies and numerical results have also considered various LD scenarios to illustrate that the bias in heritability estimation depends on the underlying LD pattern and is often small. In many cases, the standard practice of pruning markers to reduce LD (Calus and Vandenplas 2018) may be unnecessary.

In the case where $\Sigma^{-1}$ can be computed (*M *<* n*), Dicker (2014) proposes a heritability estimator (Dicker-1) that can account for the LD among markers by rotating the genotypes. The derivation of the consistency of the estimator, however, relies on the Normality assumption. In case of large *n* and *M* (M≫n), our simulation studies and analytical derivation in *Equivalence of HE and Dicker-2* show that the Dicker-2 estimator (fixed-SNP-effect model-based estimator) and HE estimator (random-SNP-effect model-based estimator), are essentially the same. Hence, in the situation M≫n, Dicker-2 estimator has limited ability to correct for the LD among markers because the HE estimator has bias under some forms of LD, as shown in Equation (13). This is a contradiction to the claim that the Dicker (2014) always provides an improved estimator of heritability in presence of LD among markers.

Further estimators have been proposed in, for example, Hou *et al.* (2019), which proposes the $h_{\mathrm{GRE}}^{2}$ estimator. This estimator’s goal is to relax assumptions about the LD structure of the data by giving each causal effect its own SNP-specific variance and has been shown to provide some robustness to LD structures. In the case that the LD matrix is estimable (*n *>* M*), if no binning is used, the $h_{\mathrm{GRE}}^{2}$ estimator is approximately equivalent to the Dicker-1-Σ estimator if n→∞ and *M* remains constant (Supplementary Section 5), but expands the scope of the Dicker-1-Σ estimator by using a pseudoinverse. This allows the $h_{\mathrm{GRE}}^{2}$ estimator to be used in cases when some markers are in perfect LD, which was not possible with the Dicker-1-Σ estimator. The $h_{\mathrm{GRE}}^{2}$ estimator also corrected bias in the Dicker-1-Σ estimator in our simulations for a finite number of individuals. Furthermore, we found that in some cases, the $h_{\mathrm{GRE}}^{2}$ estimator has lower variance than the Dicker-1 estimator even if there is no LD (Supplementary Fig. 6). This is possibly due to the use of the empirical Σ in $h_{\mathrm{GRE}}^{2}$ estimator which may reduce the variance of the estimate. We note, however, that the $h_{\mathrm{GRE}}^{2}$ estimator is not defined if *q *=* n*, where *q* is the rank of Σ (Supplementary Section 5). This situation may arise in the case that *n *<* M*. We did not study $h_{\mathrm{GRE}}^{2}$ in detail because we aimed to analytically understand the simple estimators (estimators without any binning or weighting).

Another estimator that demonstrated robustness to some forms LD was proposed in Pazokitoroudi *et al.* (2020). This estimator aimed to expand upon the HE estimator by allowing partitioning of heritability to multiple variance components. These partitioning methods can be ad hoc, but have been shown to improve robustness of estimators to MAF and LD in some cases (Evans *et al.* 2018). In the case that the genome is not partitioned, this estimator reduces to the HE estimator (Supplementary Section 6). We did not consider partitioning in this article so that we would be able to more easily understand the estimators analytically.

Fixed-SNP-effect model-based estimators generally assume that sampled individuals are independent. These approaches do not accommodate related individuals in the heritability estimation. We demonstrate that even in the absence of LD, the Dicker-1 is severely biased in the presence of related individuals. However, because of its equivalence to the HE estimator, the Dicker-2 estimator generates consistent estimates of heritability with related individuals in the absence of LD.

The likelihood-based approaches from the random-SNP-effects model category, especially the LDAK approach showed more bias under certain model misspecification as compared to the MOM estimators. Under different LD structures, the traditional GCTA approach showed more stability in terms of both bias and precision over the LDAK estimator. We did not observe any specific advantage of adjusting for LD by using the LDAK estimator.

Under the assumption of independence of individuals, the standard errors of the heritability estimator increase with the number of causal markers. This is an artifact of the assumption of complete independence (unrelatedness) of individuals. In any real sample, regardless of the extent of correction for population structure, there will always be variation in the degree of relatedness of individuals, and the extent of variation would depend on the nature of relatedness present in the sample. As shown in Simulation 3, the precision of the heritability estimators improves if we include relatives in the sample. The MSE of the estimators was generally lower when we had certain relatedness present in the sample. Moreover, the impact of increasing the number of markers on MSE was significantly less pronounced if we had relatedness in the sample. Hence, we highly recommend to at least include second cousins, if present in the study sample, in the SNP heritability estimation. If the study sample has substantial number of first cousins, it may be beneficial to assess the sensitivity of the heritability estimate after inclusion of first cousins.

In general, MOM estimators had much larger standard errors compared to the likelihood-based estimators. However, the computational gain of these MOM estimators over the likelihood estimators is significant for large *n* and *M* and often outweighs limitation of large standard error (Lin *et al*. 2022). There was no apparent bias in these estimators besides the repeat structure in Simulation 1C. For repeat structures of the causal markers, we observed underestimation in HE regression and a small upward bias for GCTA estimator.

## Data availability

The code used to generate the data is available at https://github.com/alantmin/heritability.

Supplemental material is available at *G3* online.

## Supplementary Material

jkac134_Supplementary_Data
