# Supplementary material for: Comparing heritability estimators under alternative structures of linkage disequilibrium
Source: G3 (Bethesda). 2022 Jun 8;12(8):jkac134. doi: 10.1093/g3journal/jkac134 (PMC9339317; doi:10.1093/g3journal/jkac134)
Supplement: jkac134_Supplementary_Data [file jkac134_supplementary_data.pdf]

Supplementary material

S1 Likelihood-based Approaches:

The **GCTA REML** (Yang et al., 2011) estimator is derived by assuming that random-SNP-effects $\boldsymbol{\beta} \sim N(0, \sigma_g^2 \boldsymbol{I}_{m\times m})$ and that the normalized genotypes $\boldsymbol{\Gamma}$ are fixed. It assumes a random-SNP-effect model based approach for generation of phenotypes, and uses a Euclidean distance kernel for GRM calculation. Using the Normality assumption of $\boldsymbol{\beta}$, the GCTA REML estimator assumes that $y \sim N(0, \sigma_g^2\Psi + \sigma_e^2 I)$ and uses a restricted maximum likelihood (REML) approach to estimate σ_g^2 and σ_e^2. Recently, binning methods, such as in GCTA-LDMS have been used to apply GCTA on markers binned for different linkage disequilibrium (LD) structures or for different allele frequencies (Yang et al., 2015). However, such binning techniques are somewhat adhoc and are not incorporated in our simulation and analytical derivations.

The **LDAK** (Speed et al., 2012, 2017) estimator uses a similar approach to the GCTA REML estimator, also assuming fixed genotypes and random $\boldsymbol{\beta}$. The LDAK model tries to correct for uneven LD by computing a reweighted GRM as in Equation (S1).

$$X_{ij} = (G_{ij} - 2f_j) \times [2f_j(1-f_j)]^{\alpha} \tag{S1}$$

The value $\alpha = -1.25$ is reported to generally work well with genomewide LD structure. Each of the raw genotypes is then weighted by substituting each column of G_j with $w_j G_j$, where w_j is chosen so that

$$w_j + \sum_{j}^{\prime} w_{j'} r_{jj'}^2 e^{-\lambda d_j j'} \tag{S2}$$

is constant over j. The squared correlation coefficient between SNPs j and j' is denoted by $r_{jj'}^2$, the genomic distance is denoted by $d_{jj'}$, and λ is a constant. Note that $\alpha = -1$ corresponds with the GCTA REML estimator if all w_j are 1.

S2 Method of Moments Estimators: no-LD

In this section we derive basic moment properties of the random-SNP-effect Haseman-Elston (HE) estimator and the fixed-SNP-effects Dicker-1 estimator in the case of no LD. We see their differences, but also their similarity in practice. The more general case with LD is considered in Section 2.3 of the main paper.

S2.1 Haseman Elston Method of Moments Estimator:

The HE estimator is a second-order moments estimator based on a regression of products of phenotypes $y_i y_k$ for all pairs $i \neq k$ on the corresponding (i, k) terms of the $n \times n$ GRM matrix $\mathbf{\Psi} = M^{-1}\mathbf{\Gamma}_A\mathbf{\Gamma}'_A$. Given the standardized genotypes $\mathbf{\Gamma}$, the phenotypes depend only on the first m causal markers and $\mathbf{y} = \mathbf{\Gamma}_C\ \boldsymbol{\beta} + \boldsymbol{\epsilon}$, where the independent variables $\beta_j \sim N(0, \sigma_g^2/m)$, and $\epsilon_i \sim N(0, \sigma_e^2)$.

We first consider the estimator as a regression estimate conditional on Ψ. Noting $i \neq k$, so $\mathrm{E}(\epsilon_\mathrm{i}\ \epsilon_\mathrm{k}) = 0$ and that the β_j are independent, with mean 0 and variance σ_g^2/m,

$$\mathrm{E}(\mathrm{y_i y_k}\Psi_\mathrm{ik}) = \mathrm{E}\left((\sum_{\mathrm{j}=1}^{\mathrm{m}} \Gamma_\mathrm{ij}\beta_\mathrm{j})\ (\sum_{\ell=1}^{\mathrm{m}} \Gamma_{\mathrm{k}\ell}\beta_\ell)\Psi_\mathrm{ik} \right) = \mathrm{E}\left(\sum_{\mathrm{j}=1}^{\mathrm{m}} \Gamma_\mathrm{kj}\Gamma_\mathrm{ij}\mathrm{E}(\beta_\mathrm{j}^2)\Psi_\mathrm{ik} \right) = \Psi_\mathrm{ik}^2\ \sigma_\mathrm{g}^2$$

Summing over all $n(n-1)/2$ pairs of distinct individuals, we have the method-of-moments equation

$$S_{Y\Psi} \quad \equiv \quad \sum_k \sum_{i<k} y_i y_k \Psi_{ik} \quad = \quad \sigma_g^2 \sum_k \sum_{i<k} \Psi_{ik}^2 \quad \equiv \quad \sigma_g^2 S_{\Psi\Psi}$$

so that σ_g^2 may be estimated as

$$\widetilde{\sigma_g^2} \quad = \quad \frac{S_{Y\Psi}}{S_{\Psi\Psi}} \quad = \quad \frac{\sum_k \sum_{i<k} y_i y_k \Psi_{ik}}{\sum_k \sum_{i<k} \Psi_{ik}^2} \tag{S3}$$

Then an estimate of heritability is given by dividing by the empirical variance of $\mathbf{y}$.

Here we focus on the estimate of σ_g^2 and on the numerator and denominator denoted $S_{Y\Psi}$ and $S_{\Psi\Psi}$ respectively. We consider not only the conditional model, but also the variation in Ψ over samples of genotypes from the population. Note that

$$\Psi_{ik} = M^{-1}\sum_{j=1}^{M} \Gamma_{ij}\Gamma_{kj} \quad \text{and} \quad \mathrm{E}(\Gamma_\mathrm{ij}) = 0, \;\; \mathrm{E}(\Gamma_\mathrm{ij}^2) = 1$$

So if individuals are independent, $\mathrm{E}(\Psi_\mathrm{ik}) = 0$, and if markers are independent,

$$\mathrm{E}(\Psi_\mathrm{ik}^2) = \mathrm{var}(\Psi_\mathrm{ik}) = \mathrm{M}^{-1}\mathrm{var}(\Gamma_\mathrm{ij}\Gamma_\mathrm{kj}) \;\; = \;\; \mathrm{M}^{-1}(\mathrm{E}(\Gamma_\mathrm{ij}^2))^2 \;\; = \;\; 1/\mathrm{M}$$

and, under independence of individuals i, k and independence of markers j, w, ℓ,

$$\begin{aligned}
\mathrm{E}(\mathrm{y_i y_k \Psi_{ik}}) &= M^{-1}\ \mathrm{E}\left((\sum_{\mathrm{j=1}}^{\mathrm{m}} \Gamma_{\mathrm{ij}}\beta_{\mathrm{j}} + \epsilon_{\mathrm{i}})(\sum_{\mathrm{w=1}}^{\mathrm{M}} \Gamma_{\mathrm{iw}}\Gamma_{\mathrm{kw}})(\sum_{\ell=1}^{\mathrm{m}} \Gamma_{\mathrm{k}\ell}\beta_{\ell} + \epsilon_{\mathrm{k}}) \right) \\
&= M^{-1}\mathrm{E}\left(\sum_{\mathrm{j=1}}^{\mathrm{m}} \beta_{\mathrm{j}}^2 (\sum_{\mathrm{w=1}}^{\mathrm{M}} \Gamma_{\mathrm{ij}}\Gamma_{\mathrm{iw}}\Gamma_{\mathrm{kw}}\Gamma_{\mathrm{kj}}) \right) \\
&= M^{-1}\mathrm{E}\left(\sum_{\mathrm{j=1}}^{\mathrm{m}} \beta_{\mathrm{j}}^2 \Gamma_{\mathrm{ij}}^2 \Gamma_{\mathrm{kj}}^2 \right) \quad = \quad \mathrm{M^{-1} m(\sigma_g^2/m) = \sigma_g^2/M}
\end{aligned}$$

Hence $S_{\Psi\Psi}$ has expectation $n(n-1)/2M$ and $S_{Y\Psi}$ has expectation $\sigma_g^2 n(n-1)/2M$. Empirical simulations (not shown) showed that while the standard deviation of $S_{\Psi\Psi}$ is approximately n/M, that of $S_{Y\Psi}$ is of order $n/\sqrt{M}$, but both decrease to 0 as $M \to \infty$. Thus as $M \to \infty$ with n remaining fixed, both $S_{Y\Psi}$ and $S_{\Psi\Psi}$ converge in probability to 0. As the number of markers increases, the coefficient of variation of $S_{\Psi\Psi}$ remains constant, but that of $S_{Y\Psi}$ increases, and the empirical study shows the the standard deviation of the estimate of σ_g^2 to be of order $\sqrt{M}/n$. This result is in agreement with the theoretical equations for the estimator of Dicker (2014) in the case of no LD: see Lemma 2 and the Remarks following in that paper. That is, uncertainty in σ_g^2 and hence in h^2 increases as the number of markers M increases.

S2.2 The Dicker-1 fixed-SNP-effects model moments estimator

The Dicker-1 estimator (Dicker, 2014) is also a method of moments estimator, but starts from very different assumptions. The standardized genotypes Γ_{ij} are are assumed to be distributed $N(0,1)$, independent over individuals i. The effects β_j are fixed effects, and in our case where only the first m markers are causal, $\beta_j \equiv 0$ for $j = (m+1), ...M$. The parameter to be estimated is $\sigma_g^2 \equiv \boldsymbol{\beta}'\boldsymbol{\Sigma}^*\boldsymbol{\beta}$ where here $\boldsymbol{\beta}$ is the m-vector of effects at causal markers augmented by $(M-m)$ zeros and $\boldsymbol{\Sigma}^*$ is the true LD matrix of correlations among all M markers. Because of the Normality assumption for genotypes, these can be rotated to orthonormality. This implies that the case of known $\boldsymbol{\Sigma}^*$ is mathematically equivalent to $\boldsymbol{\Sigma}^* = \boldsymbol{I}$. For simplicity we consider this case, then $\boldsymbol{\beta}'\boldsymbol{\Sigma}^*\boldsymbol{\beta} = \sum_{j=1}^m \beta_j^2$ and $m^{-1}\sum_{j=1}^m \beta_j^2 \ \equiv \ \sigma_g^2/m$, equivalent, for large m to the random-SNP-effects HE assumption $\beta_j \ \sim \ N(0, \sigma_g^2/m)$.

Dicker (2014) uses the quadratic forms $\|\mathbf{y}\|^2 = \mathbf{y'y}$ and $\|\mathbf{\Gamma}'_A\mathbf{y}\|^2 = M\ \mathbf{y}'\mathbf{\Psi}\mathbf{y}$. Without making Normality assumptions, we can compute

$$\begin{aligned} \mathrm{E}(\mathrm{M}\ \mathbf{y}'\mathbf{\Psi}\mathbf{y}) &= \sum_{i=1}^{n}\sum_{k=1}^{n} \mathrm{E}(\mathrm{M}\ \mathrm{y_i}\Psi_{\mathrm{ik}}\mathrm{y_k}) \\ &= \sum_{i=1}^{n}\sum_{k=1}^{n} \mathrm{E}\left((\sum_{\mathrm{j=1}}^{\mathrm{m}} \Gamma_{\mathrm{ij}}\beta_{\mathrm{j}} + \epsilon_{\mathrm{i}})(\sum_{\mathrm{w=1}}^{\mathrm{M}} \Gamma_{\mathrm{iw}}\Gamma_{\mathrm{kw}})(\sum_{\ell=1}^{\mathrm{m}} \Gamma_{\mathrm{k}\ell}\beta_{\ell} + \epsilon_{\mathrm{k}}) \right) \end{aligned} \tag{S4}$$

Under independence of Γ_{iw} and Γ_{kw} for $i \neq k$, the coefficient of σ_e^2 is seen to be Mn. Under independence of markers indexed by j, ℓ and w, the majority of terms in β_j and β_l in this expression disappear, leaving only a coefficient of $\sigma_g^2 = \sum_{j=1}^{m} \beta_j^2$. The remaining terms have $j = \ell \neq w$ (in which case $i = k$), or $j = \ell = w$ (in which case terms with both $i = k$ and $i \neq k$ remain). Grouping these two sets of terms this coefficient reduces to

$$(M-1)\ \mathrm{E}(\sum_{\mathrm{i}} \Gamma_{\mathrm{ij}}^2\Gamma_{\mathrm{iw}}^2) + \mathrm{E}(\sum_{\mathrm{i}}\sum_{\mathrm{k}} \Gamma_{\mathrm{ij}}^2\Gamma_{\mathrm{kj}}^2) \quad = \quad \mathrm{n(M-1) + Kn + n(n-1)} \ = \ \mathrm{n(M+n+K-2)}$$

where $K = \mathrm{E}(\Gamma_{\mathrm{ij}}^4)$. Combining the following two equations,

$$\begin{aligned} \mathrm{E}(\mathrm{n}^{-1}\mathrm{M}\ \mathbf{y}'\mathbf{\Psi}\mathbf{y}) &= (M+n+K-2)\sigma_g^2 \ + \ M\sigma_e^2 \\ \mathrm{E}(\mathrm{n}^{-1}\mathbf{y'y}) &= \sigma_g^2 + \sigma_e^2 \end{aligned}$$

and assuming $K = 3$ we obtain the Dicker (2014) method-of-moments estimator of σ_g^2:

$$\tilde{\sigma}_g^2 \ = \ (n(n+1))^{-1}(M\ \mathbf{y}'\mathbf{\Psi}\mathbf{y} \ - \ M\mathbf{y'y}) \ = \ (n(n+1))^{-1}(\|\mathbf{\Gamma}'_A\mathbf{y}\|^2 \ - \ M\|\mathbf{y}\|^2) \tag{S5}$$

Note that whereas the numerator and denominator of the HE estimator (6) always has the correct expectations, Equation (S5) is only exact if $K = 3$. Since K appears only in the term $(M+n+K-2)$ the impact will be small for large M and/or n, but it is worth noting that K can be quite large (> 100) for loci with rare alleles (see Figure S1). Under the $N(0,1)$ assumption, Dicker (2014) gives also many other expressions for high-order moments of these estimators. However, these depend more critically on the higher-order moments of the Γ_{ij}, and hence his Normality assumption.

Although the assumptions underlying the MoM estimator (S5) are very different from those of the HE estimator of Equation (6), operationally and in performance the estimators are quite similar, in the case of

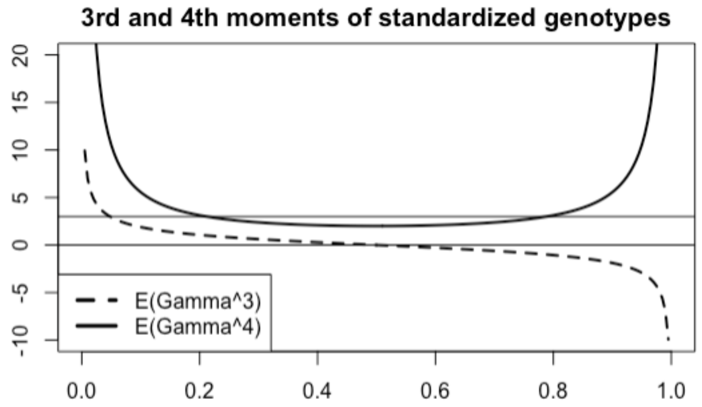

Figure S1: Skewness and kurtosis of the normalized genotypes as a function of allele frequency

known or no LD. The key difference from the HE estimator is then that whereas the latter considers only Ψ_{ik} for $i \neq k$, the Dicker estimator uses the full $n \times n$ matrix $M\mathbf{\Psi} = \mathbf{\Gamma}_A\mathbf{\Gamma}'_A$. This use of the diagonal terms Ψ_{ii} permits an estimators of σ_g^2 and σ_e^2 that is linear in the relevant quadratic forms, rather than the ratio S_{YT}/S_{TT}, but strict correctness and moment properties are dependent on the Normality assumption for Γ_{ij}.

S3 Moment based estimators: LD case

S3.1 Biases in HE estimator in the presence of LD

In the presence of LD, the HE estimator may be biased. A formula for this bias, approximating the expectation of a ratio by the ratio of expectations, is derived in Section 2.3 of the main paper. We here include some further analyses of the theoretical predictions of Equation13, that LD changes the expectation of the HE estimator by a factor of $\frac{M}{m}\frac{R_{CC}+R_{CF}}{R_{CC}\ +\ 2\ R_{CF}\ +\ R_{FF}}$.

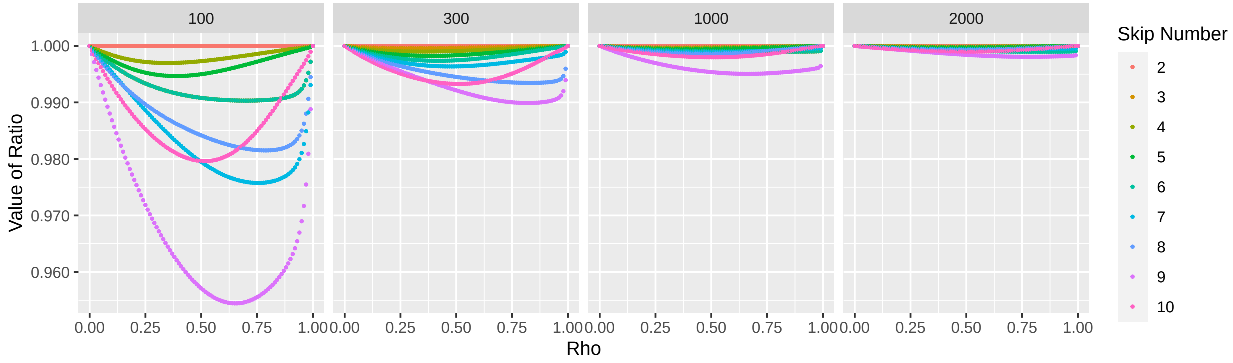

Figure S2: For the autocorrelation structure, values of the factor of Equation (Y-axis) 13 are plotted for different values of M (different panels), ρ (X-axis), and skip number (colors)

In Supplementary Figure S2, we calculated approximate theoretical biases of HE estimator in autocorrelated data for different values of ρ (x-axis), number of markers total markers M (different panels), and different "skip" numbers using equation 2.3.1. The skip number is the number of elements until a causal marker is seen. For example, if the skip number for is 2, then every second marker is causal, and all others are noncausal. The value of the ratio reported (Y-axis) indicates that the estimator is unbiased when the ratio is 1. We observed that as predicted in Section 2.3.1, no bias is observed when the skip number is 2, and furthermore, the bias is close to 1 whenever M is large for all skip numbers up to 10.

For the block structure, we can analytically show that for any number of blocks and any value of ρ, there is no bias resulting from Equation 13. We begin by computing for the case that there is 1 block consisting of all M markers, with m of the markers being causal. The correlation between all of the markers in the block is ρ. Without loss of generality, we can assume that all of the causal markers are listed before the noncausal markers. When this is the case, we can calculate that $R_{CC} = m + m(m-1)\rho$, $R_{CF} = m(M-m)\rho$,and $R_{FF} = M - m + (M-m)(M-m-1)\rho$. Substituting these values, we reach

$$E(\tilde{\sigma}_g^2) \approx \sigma_g^2 \frac{M}{m} \frac{m + m(m-1)\rho + m(M-m)\rho}{m + m(m-1)\rho + 2m(M-m)\rho + M - m + (M-m)(M-m-1)\rho}$$

and upon simplifying this expression, we find that $E(\tilde{\sigma}_g^2) \approx \sigma_g^2$. To extend this to the case of multiple identical blocks, we note that each of R_{CC}, R_{CF}, and R_{FF} are multiplied by the number of blocks, and hence the bias is the same

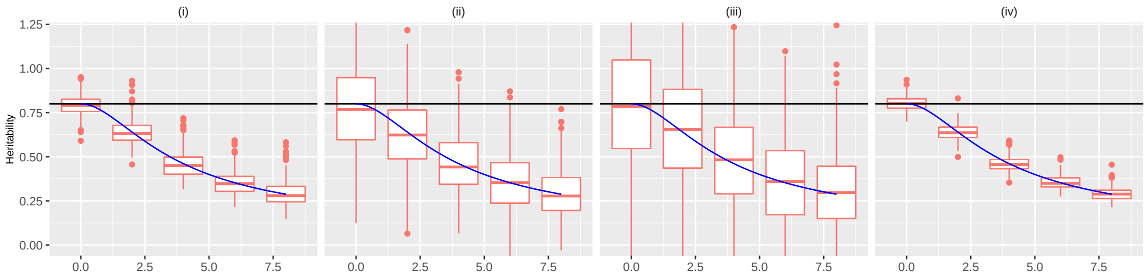

Figure S3: Estimates of h^2 (y-axis) for different values of r, the number of times that 10% of the markers are being repeated. Estimates are made using the HE estimator (red box plots) with data simulated from the repeat structure of simulation study 1. The true simulated heritability was 0.8 (solid black line). The solid blue line plots the theoretical estimates based on Equation (14). The set up is the same as in Figure 3, with (i) $n = 1000, m = 200$ (ii) $n = 200, m = 1000$, (iii) $n = 200, m = 3000$ (iv) $n = 2000, m = 1000$.

Of the simulation study examples of this paper, the bias is marked in the case of non-causal markers that repeat the genotypes of causal markers. Figure S3 aims to validate the formula for the bias and assess the variation in bias across realizations by taking estimates of heritability from the repeat structure simulated

data in simulation study 1 and comparing against the theoretical values from Equation (14). It is shown that there is close alignment of the theoretical values and the observed. We also note that the theoretical bias does not depend on the value of m, since if we multiply m by a constant c, then if we have set a fixed percentage of markers to be repeated, d is also multiplied by our constant c, and

$$\sigma_g^2 \frac{(cm+rcd)^2}{cm(cm+rcd(2+r))} = \sigma_g^2 \frac{(m+rd)^2}{m(m+rd(2+r))}$$

S3.2 Impact of LD on the Dicker-1 Estimator

We consider now the estimator of Equation (7) in the presence of LD. Although this estimator would likely not be used in practice because it does not attempt to adjust for LD and hence has a different estimand than σ_g^2 as defined here, it provides important motivation for the Dicker-2 estimator (Equation 9). We here provide justification for poor performance of the Dicker-1 estimator in simulation. Even in the absence of LD, this estimator of σ_g^2 is unbiased only if $\mathrm{E}(\Gamma_{\mathrm{ij}}^4) = 3$ (Supplementary Section S2.2) but the bias is negligible for large M or n.

Recall that for the Dicker-1 estimator (Equation 7), $\tilde{\sigma}_g^2 = (n(n+1))^{-1}(M\mathbf{y}'\mathbf{\Psi}\mathbf{y} - M\mathbf{y}'\mathbf{y})$. We begin by analyzing the term $\mathbf{y}'\mathbf{y}$. Note that $E(\Gamma_{ij}\Gamma_{il}) = \Sigma_{jl}^*$ so that

$$\mathrm{E}(\mathrm{n}^{-1}\mathbf{y}'\mathbf{y} \mid \boldsymbol{\beta}) = n^{-1}\mathrm{E}\left((\mathbf{\Gamma}_{\mathrm{C}}\boldsymbol{\beta}+\boldsymbol{\epsilon})'(\mathbf{\Gamma}_{\mathrm{C}}\boldsymbol{\beta}+\boldsymbol{\epsilon}) \mid \boldsymbol{\beta}\right) \quad = \quad \boldsymbol{\beta}'\mathbf{\Sigma}^*\boldsymbol{\beta} + \sigma_{\mathrm{e}}^2$$

where the vector $\boldsymbol{\beta}$ contains only entries for causal markers.

The fixed-SNP-effects Dicker-1 estimator estimates $\tau^2 = \boldsymbol{\beta}'\mathbf{\Sigma}^*\boldsymbol{\beta}$ (Dicker, 2014), which may differ from the additive genetic variance $\sigma_g^2 \equiv \sum_{j=1}^m \beta_j^2/m$ in the presence of LD. However, in our simulation studies, each replicate uses β_j at causal loci $j = 1, ..., m$ that are independently generated $N(0, \sigma_g^2/m)$ and independent of the standardized genotypes Γ_{ij} (Section 2.5). Thus, over replicate simulations

$$\begin{aligned}
\mathrm{E}(\boldsymbol{\beta}'\mathbf{\Sigma}^*\boldsymbol{\beta}) &= E(\sum_{\ell=1}^m\sum_{j=1}^m \beta_\ell \Sigma_{\ell j}^* \beta_j) \\
&= (\sigma_g^2/m)\sum_{j=1}^m \Sigma_{jj}^* \\
&= \sigma_g^2
\end{aligned}$$

and hence $\mathrm{E}(\mathrm{n}^{-1}\mathbf{y}'\mathbf{y}) = \sigma_{\mathrm{g}}^2 + \sigma_{\mathrm{e}}^2$

We now analyze the $\mathbf{y}'\mathbf{\Psi}y$ term. Like with the $\mathbf{y}'\mathbf{y}$ term, we may consider expectations of the estimator

over replicates assuming that β_j $(j = 1, ..., m)$ are independent $N(0, \sigma_g^2/m)$. First, we note that $\mathbf{y}'\mathbf{\Psi}\mathbf{y} = 2S_{Y\Psi} + \sum_i y_i^2 \Psi_{ii}$ so that, from Equation (12)

$$\begin{aligned}
\mathrm{E}(\mathrm{M}\ \mathbf{y}'\mathbf{\Psi}\mathbf{y}) =&\ \mathrm{E}(2\mathrm{M}\mathrm{S}_{\mathrm{Y}\Psi} + \mathrm{M}\sum_{\mathrm{i}} \mathrm{y}_{\mathrm{i}}^2 \Psi_{\mathrm{ii}}) \\
&= n(n-1)m^{-1}\sigma_g^2\ (R_{CC} + R_{CF}) + M\mathrm{E}\left[\sum_{\mathrm{i}}(\sum_{\mathrm{j}} \Gamma_{\mathrm{ij}}\beta_{\mathrm{j}} + \epsilon_{\mathrm{i}})^2(\sum_{\mathrm{w}} \Gamma_{\mathrm{iw}}^2)\right] \\
&= n(n-1)m^{-1}\sigma_g^2\ (R_{CC} + R_{CF})\ +\ nm^{-1}\sigma_g^2 \sum_{j=1}^{m}\sum_{w=1}^{M} \mathrm{E}(\Gamma_{\mathrm{ij}}^2\Gamma_{\mathrm{iw}}^2)\ +\ \mathrm{Mn}\ \sigma_{\mathrm{e}}^2
\end{aligned}$$

In general $\mathrm{E}(\Gamma_{\mathrm{ij}}^2\Gamma_{\mathrm{iw}}^2)$ is unknown, but a lower bound on the double-sum term is the no-LD value $mK + m(M-1)$ (see Supplementary Section S2.2) while a rough approximation might be $mK + (M-1)(R_{CC} + R_{CF})$, where again $K = \mathrm{E}(\Gamma_{\mathrm{ij}}^4)$. This approximation gives the overall result

$$\mathrm{E}(\mathrm{n}^{-1}\mathrm{M}\mathbf{y}'\mathbf{\Psi}\mathbf{y}) \approx\ \sigma_g^2\ (K\ +\ \frac{(n+M-2)}{m}(R_{CC} + R_{CF}))\ +\ M\sigma_e^2$$

Combining the $M\mathbf{y}'\mathbf{\Psi}\mathbf{y}$ and $M\mathbf{y}'\mathbf{y}$ terms, we have

$$\mathrm{E}\left((\mathrm{n}(\mathrm{n}+1))^{-1}(\mathrm{M}\mathbf{y}'\mathbf{\Psi}\mathbf{y}\ -\ \mathrm{M}\mathbf{y}'\mathbf{y})\right) \approx \sigma_{\mathrm{g}}^2\ (\mathrm{n}+1)^{-1}(\mathrm{K}\ +\ \frac{(\mathrm{n}+\mathrm{M}-2)}{\mathrm{m}}(\mathrm{R}_{\mathrm{CC}} + \mathrm{R}_{\mathrm{CF}}) - \mathrm{M}).$$

Since the squared correlations $r_{j\ell}^2$ are non-negative, $(R_{CC} + R_{CF}) \geq m$ and the estimator will overestimate σ_g^2 and hence also heritability h^2. Unlike the HE estimator where the LD inflates both numerator and denominator (Equation 13), the form of the estimator (7) means that it can only be inflated by LD.

S3.3 Moment estimators designed to accommodate LD

In the case when LD must be estimated from the sample data, Dicker (2014) and Schwartzman et al. (2019) developed moment-based estimators of σ_g^2, σ_e^2, and h^2 under the fixed-SNP-effects framework.

Here we consider the estimator of Dicker (2014) in the case of LD. Again, the GRM $\Psi = M^{-1}\mathbf{\Gamma}_A\ \mathbf{\Gamma}_A'$, and LD matrix $\mathbf{\Sigma} = n^{-1}\mathbf{\Gamma}_A'\mathbf{\Gamma}_A$. If the standardized genotypes, Γ_{ij}, are marginally $N(0,1)$ and independent over i, and if $\mathbf{\Sigma}^*$ is the true positive definite correlation matrix of the Γ_{ij} over j, then ${\mathbf{\Sigma}^*}^{-1/2}\mathbf{\Gamma}_A'$ are independent

$N(0,1)$ and the estimator (S5) becomes

$$\tilde{\sigma}_g^2 = (n(n+1))^{-1}((\boldsymbol{\Sigma}^{-1/2}\boldsymbol{\Gamma}'_A\mathbf{y})'(\boldsymbol{\Sigma}^{-1/2}\boldsymbol{\Gamma}'_A\mathbf{y}) - M\mathbf{y}'\mathbf{y})$$
$$= (n(n+1))^{-1}(\mathbf{y}'\boldsymbol{\Gamma}_A\boldsymbol{\Sigma}^{-1}\boldsymbol{\Gamma}'_A\mathbf{y} - M\mathbf{y}'\mathbf{y}) \quad \text{(S6)}$$

and again $\sigma_g^2 + \sigma_e^2$ is estimated by the phenotypic variance $n^{-1}\mathbf{y}'\mathbf{y}$. More generally, as shown by Dicker (2014), if $n > M$ and $\boldsymbol{\Sigma}$ is a norm-consistent estimator of the true correlation matrix the properties and results of the non-LD estimator (S5) apply also in the LD case to the estimator (S6).

However, in most applications, M is much larger than n. and the estimator (S6) breaks down, and as shown in Dicker (2014), In this case they propose to use lower-order moments of the trace of $\boldsymbol{\Sigma} = n^{-1}\boldsymbol{\Gamma}'_A\boldsymbol{\Gamma}_A$. Specifically they define

$$\mu_1 = \frac{tr(\boldsymbol{\Sigma})}{M} \text{ and } \mu_2 = \frac{tr(\boldsymbol{\Sigma}^2)}{M} - \frac{(tr(\boldsymbol{\Sigma}))^2}{Mn} \quad \text{(S7)}$$

The estimator of σ_g^2 becomes

$$\tilde{\sigma}_g^2 = \frac{\mu_1(\boldsymbol{\Gamma}'_A\mathbf{y})'(\boldsymbol{\Gamma}'_A\mathbf{y}) - M\mu_1^2\mathbf{y}'\mathbf{y}}{n(n+1)\mu_2} \quad \text{(S8)}$$

and again $\sigma_g^2 + \sigma_e^2$ is estimated by $n^{-1}\mathbf{y}'\mathbf{y}$. For more on the theory and properties of the estimator (S8) see Dicker (2014). For the current paper, we implement this estimator as "Dicker-2" in our simulations and results.

Schwartzman et al. (2019) proposed a method of moments estimator based on that of Dicker (2014). They derive a form that depends only on summary statistics instead of the raw genotypic and phenotypic data and hence their estimator has wider applicability. However, in the basic form (not using only summary statistics) their estimator is essentially equivalent to the estimator (S8), so we do not consider it further in this paper.

S4 Simulation of Genetic Marker LD Structures

Autocorrelated: we assume that for each individual, M markers are generated from a multivariate Gaussian with $AR1(\rho)$ covariance matrix. We generate the markers for each individual independently. In other words, we assume that for individual i, genotypes $\tilde{G}_i$ are generated from $\tilde{G}_i \sim N(0, \Sigma)$, where

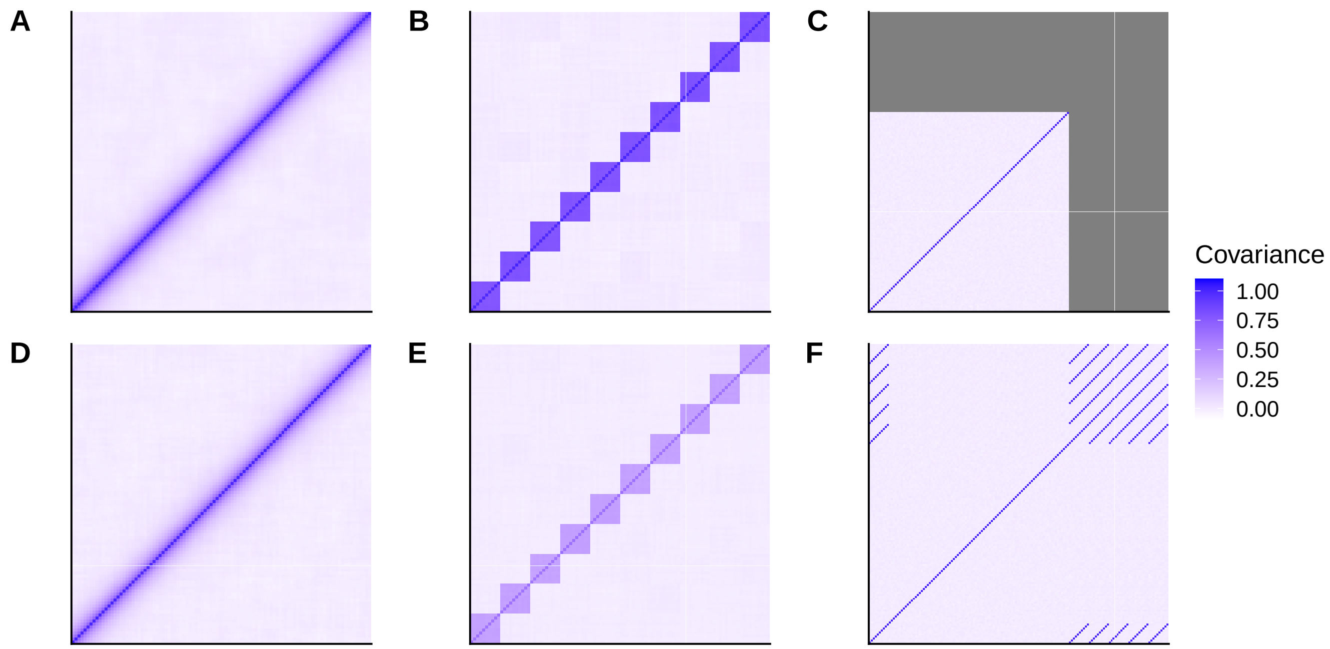

Figure S4: These panels plot the empirical covariance matrices for simulated genotypes from 10,000 individuals and $p = 100$ markers. The correlation between markers decreases after discretization but the pattern generally remains the same. (A) Autocorrelated markers were generated from the Gaussian model, i.e. plotting $Cov(\tilde{G})$ (B) Blocked markers were generated from the Gaussian model. (C) Independent markers were generated. (D) Autocorrelated markers were generated and then discretized and normalized, i.e. this is $Cov(\Gamma)$ (E) Blocked markers were discretized and normalized. (F) Repeated markers were generated with 10 markers being repeated 5 times.

$$\Sigma = \begin{pmatrix} 1 & \rho & \rho^2 & \dots & \rho^{M-1} \\ \rho & 1 & \rho & \dots & \rho^{M-2} \\ \vdots & & \vdots & & \vdots \\ \rho^{M-1} & \rho^{M-2} & \rho^{M-3} & \dots & 1 \end{pmatrix}$$

The continuous values $\tilde{G}_i$ are then converted to discrete genotypes G_i taking value 0, 1 or 2. For a marker with alternate allele frequency f, $G_{ij} = 0, 1$, or 2, depending on if $\tilde{G}_{ij}$ is less than $\Phi^{-1}(f^2)$, between $\Phi^{-1}(f^2)$ and $\Phi^{-1}(f^2 + 2f(1-f)) = \Phi^{-1}(2f - f^2)$, or greater than $\Phi^{-1}(2f - f^2)$, where $\Phi(\cdot)$ is the $N(0, 1)$ distribution function. Note that this trichotomy gives the correct marginal genotype probabilities, but reduces the genotypic correlation (LD) between markers below that used in the simulation matrix Σ: compare panels A with D, or B with E in Figure S4.

Block: we generate block genotypes according to the same mechanism as the autocorrelated genotypes, except we choose that

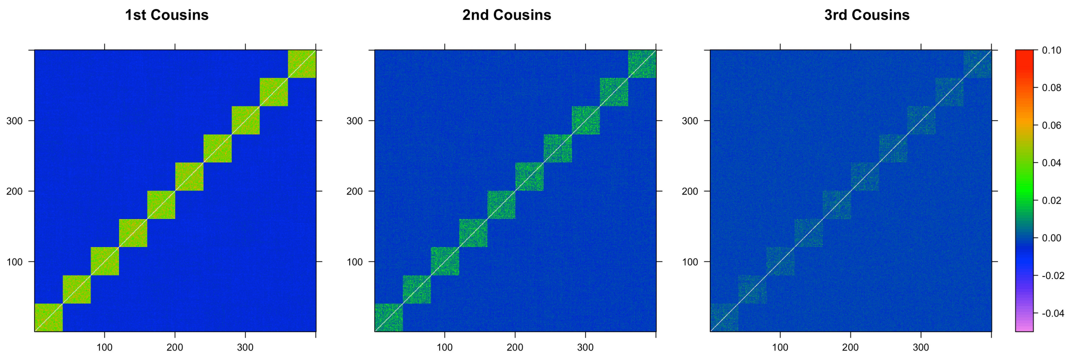

Figure S5: Colors represent values of the log of 1 plus the average of 100 GRMs generated from 400 individuals. The i, jth entry of the matrix corresponds to the relatedness between ith individual and the jth individual. Sets of cousins are adjacent in groups of 40. Colors are thresholded at 0.1, and set to white if it is above the threshold.

$$\Sigma = \begin{pmatrix} 1 & \rho & \rho & \dots & \rho \\ \rho & 1 & \rho & \dots & \rho \\ \vdots & & \vdots & & \vdots \\ \rho & \rho & \rho & \dots & 1 \end{pmatrix}$$

for each block. We assume that there are 10 blocks, each with $M/10$ markers.

Repeat: In this case m marker genotypes are independently generated from the binomial distribution. That is, for a marker with alternate allele frequency f, $G_{ij} \sim Binomial(2, f)$. We designate a proportion of markers to be repeated. We repeat these markers r times.

Choice of causal markers

For the three simulation LD structures, we selected $\boldsymbol{G}_c$ to be a subset of $\boldsymbol{G}$. For the autocorrelation and block simulated genotypes, we chose alternating markers to be causal and non-causal markers. For the repeat structure the original m markers were chosen to be causal, while the repeat genotypes were non-causal. The genotypes were standardized to each have mean 0 and variance 1, using the empirical allele frequencies in the simulated sample of n individuals. The matrix $\boldsymbol{\Gamma}_A$ of standardized genotypes was formed as given in Equation (1), while $\boldsymbol{\Gamma}_C$ is the corresponding matrix for the m causal markers.

S5 Equivalence of a simplified h^2_{GRE} and Dicker-1-Σ

Recall that from Section 2.2, the Dicker-1 estimator can be expressed as $(n(n+1))^{-1}(\|\boldsymbol{\Gamma_A}'y\|^2 - M\mathbf{y}'\mathbf{y})$ if $\boldsymbol{\Sigma}^*$ is known to be the identity matrix. In the case that $\boldsymbol{\Sigma}^*$ is known or estimable but not the identity, we

have Dicker-1-Σ. We replace $\mathbf{\Gamma}$ by $\mathbf{\Gamma}\mathbf{\Sigma}^{-1/2}$, and we have

$$\frac{M\left[(\mathbf{\Gamma}\mathbf{\Sigma}^{-1/2})'\mathbf{y}\right]'\left[(\mathbf{\Gamma}\mathbf{\Sigma}^{-1/2})'\mathbf{y}\right] - M\mathbf{y}'\mathbf{y}}{n(n+1)} = \frac{M\mathbf{y}'\mathbf{\Gamma}\mathbf{\Sigma}^{-1/2}(\mathbf{\Sigma}^{-1/2})'\mathbf{\Gamma}'\mathbf{y} - M\mathbf{y}'\mathbf{y}}{n(n+1)} \tag{S9}$$

$$= \frac{M\mathbf{y}'\mathbf{\Gamma}\mathbf{\Sigma}^{-1}\mathbf{\Gamma}'\mathbf{y} - M\mathbf{y}'\mathbf{y}}{n(n+1)} \tag{S10}$$

On the other hand, if we do not apply partitioning, the h^2_{GRE} estimator is expressed as

$$h^2_{GRE} = \frac{n\hat{\boldsymbol{\beta}}'\mathbf{\Sigma}^{-1}\hat{\boldsymbol{\beta}} - q}{n-q} \tag{S11}$$

$$\approx \frac{\mathbf{y}'\mathbf{\Gamma}\mathbf{\Sigma}^{-1}\mathbf{\Gamma}'\mathbf{y} - \mathbf{y}'\mathbf{y}q}{n(n-q)} \tag{S12}$$

$$\approx \frac{\mathbf{y}'\mathbf{\Gamma}\mathbf{\Sigma}^{-1}\mathbf{\Gamma}'\mathbf{y} - \mathbf{y}'\mathbf{y}q}{n(n+1)} \tag{S13}$$

Here, $\hat{\boldsymbol{\beta}}$ is defined to be $\frac{1}{n}\mathbf{\Gamma}'\mathbf{y}$, as per (Hou et al., 2019). Furthermore, q is the rank of $\mathbf{\Sigma}$. If $n > M$ and Γ is full rank, then $q = M$. We assume that $\mathbf{y}'\mathbf{y} \approx n$. Furthermore, for $n >> M$, we assume $n(n-1) \approx n(n-M)$. With these assumptions, Equation (S13) and Equation (S10) are the same, demonstrating the equivalence. Upon rescaling the Dicker-1-Σ estimator by $\frac{n-1}{n-M}$, the Dicker-1-Σ and the h^2_{GRE} estimator are essentially equivalent (Supplementary Figure S6)

We simulated data similarly to Section 2.5, but excluded cases where $n < M$ because if $n < M$ and $\mathbf{\Gamma}$ is rank n, then $\mathbf{\Sigma}$ can often have rank n, in which case the GRE estimator is not well defined. We excluded the repeat LD structure because we were unable to calculate the Dicker-1-Σ estimator since we were unable to calculate Σ^{-1}. We found that the h^2_{GRE} estimator was robust to the structures of LD that we presented here. Furthermore, even though when $r = 0$ or $\rho = 0$, we have that $\Sigma^* = I$, the h^2_{GRE} could have lower MSE than Dicker-1. This may be because including the empirical Σ may reduce the variance of the estimate.

S6 Equivalence of RHE-mc with one component to HE

The randomized Haseman Elston estimator with multiple components (RHE-mc) uses a system of normal equations to estimate σ_g^2 and σ_e^2 (Equation 7 in (Pazokitoroudi et al., 2020)). If only one component is used in this estimator, then the equations become

$$\begin{pmatrix} tr(\mathbf{\Psi}\mathbf{\Psi}) & n \\ n & n \end{pmatrix} \begin{pmatrix} \tilde{\sigma}_g^2 \\ \tilde{\sigma}_e^2 \end{pmatrix} = \begin{pmatrix} \mathbf{y}'\mathbf{\Psi}\mathbf{y} \\ \mathbf{y}'\mathbf{y} \end{pmatrix} \tag{S14}$$

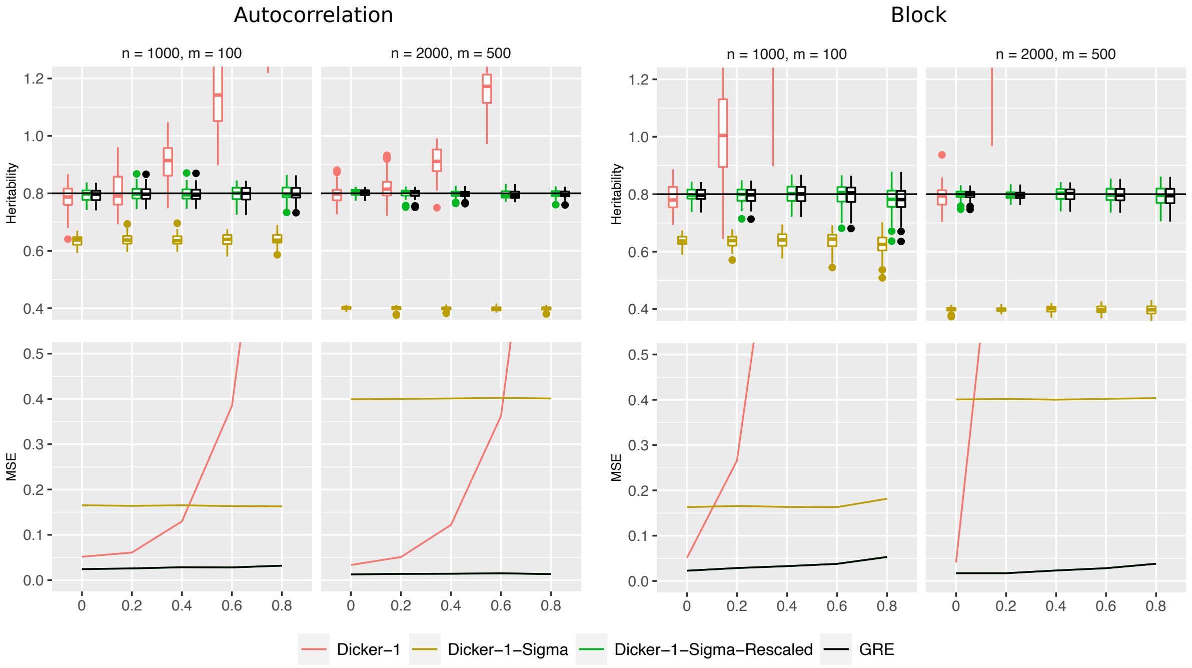

Figure S6: We simulated 50 data sets for each of autocorrelation, block, and repeat structures of each of the estimators, and including the h^2_{GRE} estimator (black). The X-axis plots ρ. A horizontal line is shown at $h^2 = .8$. On the top row, estimates of heritability are shown. On the bottom row, MSEs are shown.

Upon solving the system of equations for $\tilde{\sigma}_g^2$, we obtain the estimator

$$\tilde{\sigma}_g^2 = \frac{\mathbf{y}'\mathbf{\Psi}\mathbf{y} - \mathbf{y}'\mathbf{y}}{tr(\mathbf{\Psi}'\mathbf{\Psi}) - n} \tag{S15}$$

Note that we used the fact that $\mathbf{\Psi}$ is symmetric, and hence $\mathbf{\Psi} = \mathbf{\Psi}'$. Because $\mathbf{y}$ is standardized, we have $\mathbf{y}'\mathbf{y} = 1$. Then we note that Equation S15 is the same as Equation 15.
